# RISC-mediated control of selected chromatin regulators stabilizes ground state pluripotency of mouse embryonic stem cells

**DOI:** 10.1186/s13059-016-0952-x

**Published:** 2016-05-06

**Authors:** Luca Pandolfini, Ettore Luzi, Dario Bressan, Nadia Ucciferri, Michele Bertacchi, Rossella Brandi, Silvia Rocchiccioli, Mara D’Onofrio, Federico Cremisi

**Affiliations:** Scuola Normale Superiore of Pisa, Piazza dei Cavalieri, 7, 56124 Pisa, Italy; Department of Surgery and Translational Medicine, University of Florence, Florence, Italy; Cancer Research UK Cambridge Institute, University of Cambridge, Cambridge, CB2 0RE UK; Institute of Clinical Physiology CNR, Via Moruzzi 1, 56124 Pisa, Italy; University of Nice Sophia-Antipolis, Parc Valrose, 28 Avenue Valrose, F-06108 Nice, France; Genomics Facility, European Brain Research Institute (EBRI) “Rita Levi-Montalcini”, Via del Fosso di Fiorano 64, 00143 Rome, Italy; Institute of Translational Pharmacology, CNR, Via del Fosso del Cavaliere 100, 00133 Rome, Italy; Wellcome Trust/CRUK Gurdon Institute, Tennis Court Road, Cambridge, CB2 1QN UK; Institute of Biomedical Technologies (ITB), National Research Council (CNR) of Pisa, Via Moruzzi 1, 56124 Pisa, Italy

## Abstract

**Background:**

Embryonic stem cells are intrinsically unstable and differentiate spontaneously if they are not shielded from external stimuli. Although the nature of such instability is still controversial, growing evidence suggests that protein translation control may play a crucial role.

**Results:**

We performed an integrated analysis of RNA and proteins at the transition between naïve embryonic stem cells and cells primed to differentiate. During this transition, mRNAs coding for chromatin regulators are specifically released from translational inhibition mediated by RNA-induced silencing complex (RISC). This suggests that, prior to differentiation, the propensity of embryonic stem cells to change their epigenetic status is hampered by RNA interference. The expression of these chromatin regulators is reinstated following acute inactivation of RISC and it correlates with loss of stemness markers and activation of early cell differentiation markers in treated embryonic stem cells.

**Conclusions:**

We propose that RISC-mediated inhibition of specific sets of chromatin regulators is a primary mechanism for preserving embryonic stem cell pluripotency while inhibiting the onset of embryonic developmental programs.

**Electronic supplementary material:**

The online version of this article (doi:10.1186/s13059-016-0952-x) contains supplementary material, which is available to authorized users.

## Background

Embryonic stem (ES) cells tend to spontaneously differentiate in the absence of external inductive signals [1]. The first step of ES cell differentiation, commonly reported as “priming”, is mostly associated with changes in the dynamics of chromatin, post-translational modifications of histones, and a general remodeling of nuclear architecture [2]. Priming is considered necessary for lineage specification in the early embryo but the exact mechanisms mediating its action on the transition from pluripotency state to the differentiation of embryonic tissues are not understood. Inhibition of protein translational noise [3] and transcriptional “leakage” [4, 5] characterize mouse ES cells. This indicates that lineage specification during early embryonic development could be driven by reduction of the transcribed portion of the genome but it also poses the question of how pluripotency can accommodate the transcription of tissue-specific genes.

We speculated that a tight inhibitory control of translation is crucial to maintain pluripotency and that inhibition of protein translation through microRNA (miRNA) and the RNA-induced silencing complex (RISC) [6] might represent one strategy to avoid a “transcriptional paradox”. There is, indeed, an established body of evidence indicating that release from RISC-mediated translational inhibition, produced through the disruption of components of the miRNA maturation pathway such as Dicer [7] or DGCR8 [8], severely impairs pluripotency in ES cells. This observation implies that inhibition of protein translation is necessary for pluripotency. However, while the general involvement of RISC is established, little is known about the families of genes subject to this control. In our investigation, we found that a set of mRNAs encoding chromatin regulators is selectively released from miRNA-mediated protein translation inhibition during priming and we conclude that their inhibition is crucial for the maintenance of ground state pluripotency.

## Results

### Epiblast-like aggregate cells are equivalent to primed pluripotent cells

**Fig. 1:**
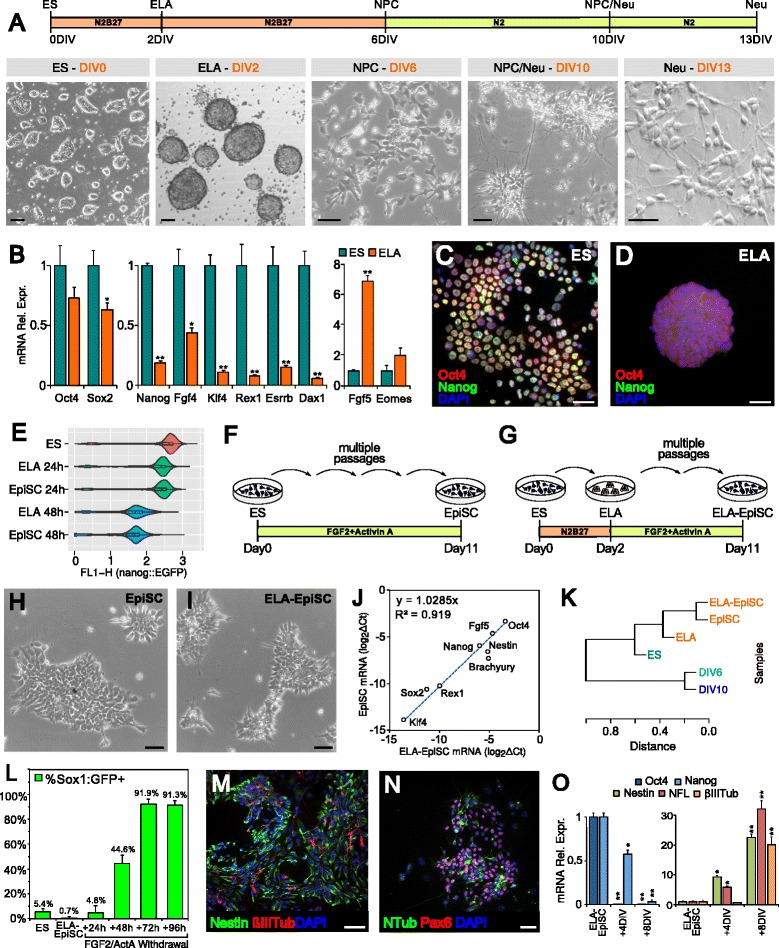
**a** ES cell in vitro neuralization. *DIV* days of in vitro differentiation. *0DIV* corresponds to the time of leukemia inhibitory factor (LIF) withdrawal. *N2* and *B27* are the supplements used in the minimal medium of differentiation. Example of bright-field microphotographs of cells at different DIV are shown on the *bottom. ELA* epiblast-like aggregates, *NPC* neural progenitor cells, *NPC/Neu* neural precursors, *Neu* differentiated neurons. **b** RT-PCR gene expression analysis. Values are relative to β-actin mRNA expression. Highest and lowest expression levels were normalized to 1 in the *left*/*middle* histograms and in the *right* histogram, respectively. **c**, **d** Oct4 and Nanog immunodetection in ES cells (**c**) or ELA cells (**d**). **e** Violin plot shows the distribution of green fluorescent protein (GFP) intensity in a TNG-A Nanog::GFP line [16] in LIF/serum (ES cells, *red*) and 24 h (*green*) or 48 h (*blue*) after LIF/serum withdrawal (*ELA*) or Activin/fibroblast growth factor (FGF)2 induction (*EpiSC*), respectively. **f**, **g** Derivation of epiblast stem cells (EpiSC) and ELA-EpiSC from ES and ELA cells, respectively. **h**, **i** EpiSC and ELA-EpiSC bright-field images. **j** Expression correlation of markers of pluripotency and priming between EpiSC (*y-axis*) and ELA-EpiSC (*x-axis*). Values are expressed as log_2_ΔCt of RT-PCR assay; *R* ^*2*^ coefficient of determination. **k** Hierarchical clustering analysis on Spearman correlation between different microarray samples. **l** Flow cytofluorimetric analysis of Sox1::GFP cells (46C line), indicating the ratio of GFP-positive cells (*y-axis*) in different cell types or times of differentiation (*x-axis*). **m**, **n** Immunodetection of neural markers at 7 days of ELA-EpiSC neuralization. **o** RT-PCR gene expression analysis as in **b** in ELA-EpiSC after 4 (*+4DIV*) or 8 (*+8DIV*) days from FGF2/Activin A withdrawal. Error bars in **b**, **l**, and **o** show standard error. In **b** and **o** *p = 0.05, **p = 0.01 (REST randomization test). Scale bars are 30 microns in **a**, **c**, and **d**, 40 microns in **h**, **i**, **m**, and **n**

**Fig. 2:**
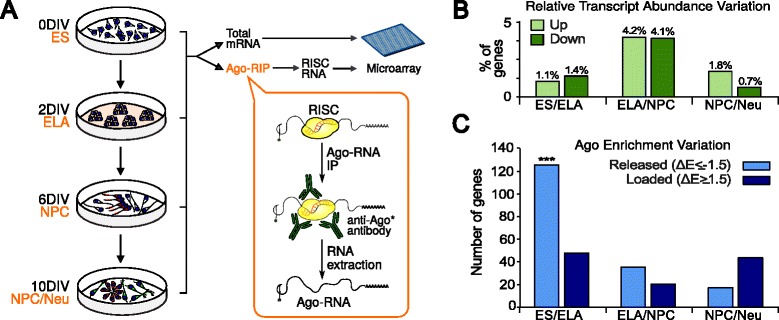
**a** ES cell in vitro neuralization and RNA analysis. *DIV* days of in vitro differentiation. **b** The fraction of total mRNAs that are up- or down-regulated between consecutive steps of differentiation, with a threshold of |log_2_FC| > 2.5 (where FC is fold change). **c** Number of genes whose mRNA is significantly released (or loaded) by Argonaute (Ago; see “Methods”); ***p = 0.001 (χ^2^-test). *IP* immunoprecipitation

To address the role of RISC in ES cell differentiation, we employed a protocol of mouse ES cell neuralization that reproduces the main steps of early embryonic neural development [9] (see “Methods”). Cells at 2, 6, 10, and 13 days of in vitro differentiation (DIV) correspond to epiblast-like aggregates (ELA), neural progenitor cells (NPC), neural precursors (NPC/Neu) and differentiated neurons (Neu), respectively (Fig. 1a). To establish the identity of ELA cells, we focused on gene expression changes at the ES–ELA transition. General markers of pluripotency, Oct4 and Sox2, were only marginally affected during the ES–ELA transition (Fig. 1b), indicating an undifferentiated condition. However, epiblast markers fibroblast growth factor (FGF)5 [10] and eomesodermin [11] were up-regulated. FGF4, Klf4, Rex1, Esrrb, and Dax1, which are markers of ground-state pluripotency [12, 13], and Nanog were highly down-regulated (Fig. 2b–d). This is similar to what is observed in post-implantation epiblast stage embryos [14] or in mouse ES cell (mESC)-derived epiblast stem cells (EpiSC) [15]. To further investigate this, we performed a more detailed analysis of Nanog expression. The distribution of green fluorescent protein (GFP) intensity of a TNG-A Nanog::GFP ES cell line [16], while shifting from high to low level during the ES–ELA transition, maintains a narrow peak and is almost superimposable on the distribution of GFP intensity during the ES–EpiSC transition (Fig. 2e); this indicates that the ES–ELA transition occurs in a quite homogeneous fashion and suggests that ELA cells might be equivalent to post-implantation epiblast cells.

Mouse EpiSC can be maintained in a pluripotent state similar to that of human ES cells (hESCs) [17, 18] by FGF2/Activin A treatment [12, 13]. Ground-state mESC cultured for several passages in FGF2/Activin-containing medium (Fig. 2f) acquire a flattened morphology (Fig. 2h) and retain a gene expression profile similar to ELA cells and consistent with epiblast identity [15, 19]. Accordingly, ELA-derived cells could be dissociated and maintained in an EpiSC-like state by adding FGF2/Activin A to culture medium (Fig. 2g). The resulting cells (ELA-EpiSC; Fig. 2i) displayed a high level of correlation with the gene expression profile of mESC-derived EpiSC (Fig. 2j, k).

As well as ground-state mESC, pluripotent stem cells derived from the epiblast spontaneously neuralize when deprived of FGF2 and Activin A. Similarly, more than 90 % of ELA-EpiSC induced the early marker of neuralization Sox1 72 h after FGF2/Activin A withdrawal (Fig. 2l). These cells became nestin-positive neural progenitor cells (Fig. 2m) and differentiating neurons positive to N-tubulin and Pax6 (Fig. 2n) by down-regulating Oct4 and Nanog and up-regulating neuronal differentiation markers (Nestin, β-III-tubulin and NFL; Fig. 2o).

These results thus indicate that ELA cells can be considered to be equivalent to epiblast stem cells and, therefore, the ES–ELA transition could be used as an in vitro model of the transition from inner mass cells to *primed* post-implantation epiblast cells. Using ELA instead of EpiSC allows the requirement for exogenous FGF and Activin to be avoided, whose action could not parallel the autocrine signaling of the developing embryo. Moreover, since ELA cells are not steady-state cultures as EpiSC are, they retain the transient nature of epiblast cells in vitro and provide a better model than EpiSC for studying spontaneous, rapidly occurring molecular changes during this developmental transition.

### Profiling of Argonaute occupancy at early steps of ES cell differentiation

We performed a global survey of the mRNAs that interact with Argonaute (Ago) during the first stages of in vitro differentiation. Towards this goal, we isolated Ago-interacting RNAs by immunoprecipitating Ago proteins (Ago* in Additional file 1: Figure S1a), which are the main RISC components [6]. Ago–RNA and total RNA from cells at 0, 2, 6, and 10 DIV were hybridized to gene expression microarrays (Fig 2a). The release of mRNA from Ago at the transition between sequential steps was evaluated as the variation of the ratio between Ago–RNA and total RNA levels after normalization (Ago Enrichment in Additional file 1: Figure S1b, c; Additional file 2: Table S1; see “Methods”). While most of the transcriptional regulation occurred at the ELA–NPC transition (Fig. 2b), the highest release of mRNA by Ago was observed at the ES–ELA transition (Fig. 2c), with more than 100 mRNA species significantly released from Ago binding. We verified that this could not be explained by either different efficiency in Ago immunoprecipitation (Additional file 1: Figure S1a) or differential expression of Ago- or miRNA-related genes in the two conditions (Additional file 1: Figure S1d). The variation in Ago enrichment was coherently correlated with the variation in total RNA level at the ES–ELA transition for 82.4 % of genes (Additional file 1: Figure S1e), confirming a predominant effect of RISC on mRNA destabilization [20].

### mRNAs of selected chromatin regulator families are preferentially released by Ago during the ES–ELA transition

**Fig. 3:**
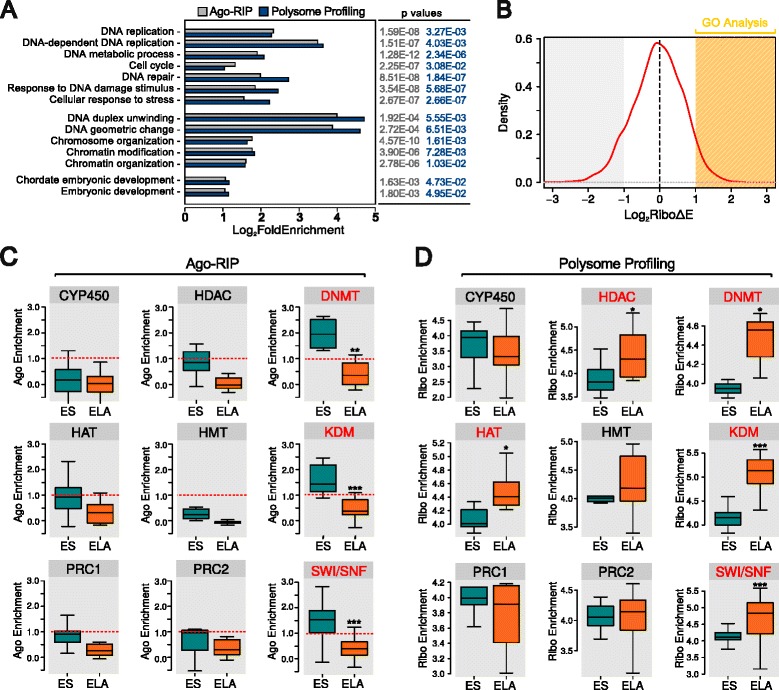
**a** GO terms significantly enriched in both gene sets of mRNAs released from Ago (Ago-RIP, *gray*) and of mRNAs significantly loaded on ribosomes (Polysome profiling, *dark blue*) at the ES–ELA transition, as obtained by DAVID (see “Methods”). Fold enrichment bars are grouped according to DNA replication, chromatin regulation, and embryonic development terms; a complete list of all enriched terms in each subset is provided in Additional file 3: Table S2. **b** Distribution of log_2_ ribosome enrichment variation (*RiboΔE*) during the ES–ELA transition. **c**, **d** Box plots of Ago enrichment (**c**) and log_2_ ribosome enrichment (*Ribo Enrichment*) (**d**) of mRNAs belonging to distinct classes of chromatin regulators, as listed in Histome [25] (for a complete list of genes taken into account see also Additional file 3: Table S2). In *red*, families showing statistically significant differences in enrichment. *HAT* histone acetyl-transferases, *HDAC* histone deacetylases, *HMT* histone methyl-transferases, *PRC1/2* Polycomb repressor complex 1/2. *p = 0.05, **p = 0.01, ***p = 0.001 (Wilcoxon test)

We investigated the nature of the mRNAs released by Ago during priming by analyzing the distribution of Gene Ontology (GO) terms in our Ago RNA immunoprecipitation (Ago-RIP) results. Genes whose mRNAs were mainly associated with Ago in ES cells belong to gene ontologies related to DNA repair, replication, chromatin organization and modification, and embryonic development (Fig. 3a; Additional file 3: Table S2). The release by Ago of mRNAs involved in DNA repair and replication in ELA cells is consistent with the marked enhancement of replication observed in epiblasts following implantation [21].

Chromatin modifications are postulated to play a crucial role in priming [22–24] but miRNA-mediated translational control of chromatin regulators has never been extensively described. Therefore, we focused our attention on the release by Ago of distinct families of chromatin modifiers [25] (Additional file 3: Table S2).

While the families of mRNAs coding for de novo DNA methyltransferases (DNMT), histone lysine demethylases (KDM), and SWItch/Sucrose NonFermentable nucleosome remodeling complex (SWI/SNF) showed a more than twofold increase of Ago enrichment in ES cells compared with ELA cells, other families of chromatin regulators showed little or no increase, similar to what was observed for families of mRNAs with unrelated functions such as cytochrome P450 (CYP450; Fig. 3c).

### Ago enrichment is predictive of protein translation activity

To detect changes in protein translation at the ES–ELA transition, we evaluated ribosome occupancy by means of polysome profiling [26, 27] (see “Methods”; Additional file 1: Figure S2a, b), adapting the translating ribosome affinity purification (TRAP) protocol for our purposes. We measured the amount of mRNAs loaded on translating ribosomes and calculated ribosome enrichment as an estimate of ribosome occupancy. Our analysis showed that the GO categories of genes showing increased ribosome loading during the ES–ELA transition were almost superimposable on those found for genes released from Ago (Fig. 3a, b). Strikingly, DNMT, KDM, and SWI/SNF families showed a ribosome enrichment essentially reciprocal to their Ago enrichment (Fig. 3d).

**Fig. 4:**
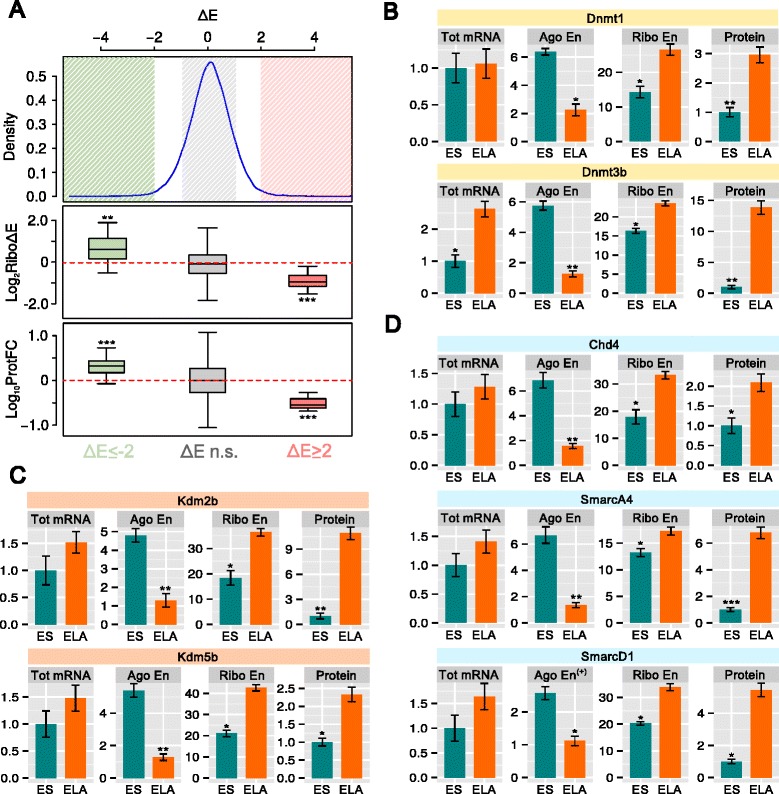
**a** *Upper panel*: the distribution of Ago enrichment variation (*ΔE*) during the ES–ELA transition. The most negative side of the *x-axis* (ΔE < −5), containing few values (density < −0.002), is not shown for clarity (for comparison, see Additional file 1: Figure S1e). *Middle* and *lower panels*: comparison between log_2_ variation of ribosome enrichment (*RiboΔE*, *middle panel*) or log_10_ protein fold changes (*lower panel*) of three subsets of genes displaying significant Ago release (ΔE ≤ −2, *green*), significant Ago loading (ΔE ≥ 2, *pink*) or non-significant change of Ago enrichment (−1 ≤ ΔE ≤ 1, *gray*). *Asterisks* indicate the *p* value (Student’s *t*-test, ***p = 0.001) against the null hypothesis of mean equal to 0. **b**–**d** Total mRNA fold change (*Tot mRNA*), linear Ago enrichment (*Ago En*), ribosome enrichment (*Ribo En*), and MS fold change (*Protein*) for the members of DNMT, KDM, and SWI/SNF classes of chromatin regulators detected in ES and ELA nuclear cell extracts. The *plus sign* marks values that are slightly out of the ranges indicated in “Methods” but are still relevant. Other genes unrelated to these families but displaying similar regulation are listed in Additional file 4: Table S3. **p = 0.01, ***p = 0.001 (Wilcoxon test). Error bars show standard error

To further verify our findings, we carried out label-free mass spectrometry (MS) of protein nuclear extracts. Notably, we observed a general correspondence of protein level variation with both Ago-RIP and polysome profiling data (Fig. 4a). This observation indicates that our method of evaluation of Ago enrichment is quite predictive of protein translation inhibition. Using MS, we identified two DNMT members (Dnmt1 and Dnmt3b), two KDM members (Kdm2b and Kdm5b), and three SWI/SNF members (Chd4, SmarcA4, and SmarcD1) as significantly increased during the transition to ELA (Additional file 4: Table S3). In all these cases, the increase in overall mRNA level was modest, strongly indicating a post-transcriptional effect (Fig. 4b–d). Dnmt3b, SmarcA4, and Kdm2b proteins were barely detectable in the cytoplasm of ES cells (Additional file 1: Figure S2E), arguing against a switch in cytoplasmic to nuclear localization as an explanation for the protein changes observed. Indeed, this phenomenon could most likely be explained by the dramatic drop in Ago enrichment displayed by these specific mRNA families during the ES–ELA cell transition. Furthermore, it would likely not be due to a general drop in RISC activity as the bulk of variation of Ago enrichment is symmetrical and zero-centered (Fig. 4a, upper panel). Intriguingly, the modest increase in total mRNA levels suggests either that these specific mRNAs undergo a lower RISC-mediated decay than that of the majority of Ago-loaded mRNAs (see above) or that an additional transcriptional modulation is occurring.

### DNMT, KDM, and SWI/SNF activities are necessary to switch between ground and primed pluripotent cell states

**Fig. 5:**
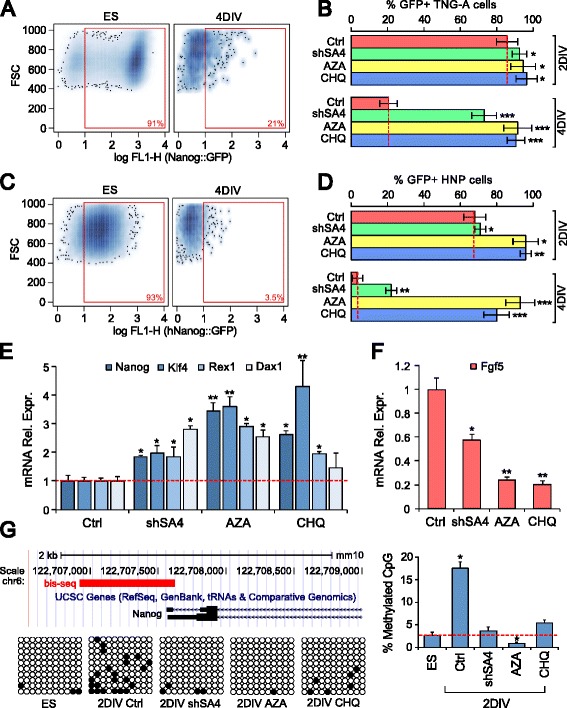
**a** Flow cytofluorimetry analysis of TNG-A Nanog::GFP cells at 4 DIV. **b** GFP-positive cell ratios at 2 DIV and 4 DIV after inhibition of SmarcA4 (*shSA4*), DNMT (*AZA*), or KDM (*CHQ*) compared with control (*Ctrl*). **c, d** Similar analysis as in **a** and **b**, with a mESC line carrying GFP under the human Nanog promoter (*HNP*). **e**, **f** RT-PCR gene expression analysis. Values are relative to β-actin mRNA expression. The lowest and highest expression levels were normalized to 1 in the *left* and *right* histograms, respectively. **g** Nanog proximal promoter methylation. The scheme shows the region of the mouse Nanog promoter amplified for bisulfite-treated DNA sequencing (bis-seq). *Black circles* in grid rows indicate CpG methylation sites. The *histogram* shows the percentage of CpG methylation in different culture conditions as above. *p = 0.05, **p = 0.01, ***p = 0.001 (**b**, **d**, **g**, Student’s *t*-test; **e**, **f**, REST randomization test). Error bars show standard error

We assayed the impact of the functional inhibition of chromatin regulators on the expression of Nanog, which is down-regulated during priming [28]. Using the mouse TNG-A Nanog::GFP line [16] as a model, we inhibited our target genes using either drugs, 5-azacytidine (AZA) [29] for DMNT and 5-carboxy-8-hydroxyquinoline (CHQ) [30] for KDM, or lentiviral-mediated transduction of short hairpin RNAs (shRNA) against SmarcA4 (shSA4) and measured the effect on Nanog expression at 0, 2, or 4 DIV (Fig. 5a, b; Additional file 1: Figure S3a–c). AZA, CHQ, and shSA4 significantly increased the ratio of GFP-positive cells at 4 DIV compared with control (Fig. 5b), maintaining a high median content of fluorescence (Additional file 1: Figure S3c). A similar analysis was performed using a mESC line carrying GFP under the control of the human Nanog promoter (HNP; Additional file 1: Figure S3d–g). This analysis produced similar results (Fig. 5c,d; Additional file 1: Figure S3c), suggesting that DNMT, KDM, and SWI/SNF activity is necessary for human Nanog down-regulation during priming and that such a requirement might have been conserved in mice and humans. In addition to Nanog, markers of pluripotency such as Klf4, Rex1, and Dax1 were up-regulated in cells treated with AZA, CHQ, or shSA4 compared with control at 4 DIV, while the priming marker Fgf5 was down-regulated (Fig. 5e, f).

We investigated the role of DNMT in silencing Nanog expression more directly by monitoring Nanog promoter methylation, which was consistently inhibited by AZA treatment at 2 DIV compared with control (Fig. 5g). Interestingly, CHQ treatment and shSA4 transduction blocked the methylation of the Nanog promoter as well (Fig. 5g), indicating the requirement of KDM and SWI/SNF activities in this modification, in agreement with previous reports [31]. At 2 DIV, cells treated with AZA and CHQ showed a global mRNA expression profile almost identical to that of ES cells, as evaluated by hierarchical clustering and fold change analysis (Additional file 1: Figure S3h–n). Cells transduced with shSA4 showed a modulation of their profile consistent with that of AZA and CHQ (Additional file 1: Figure S3l) but, in this case, gene expression clustered with that of ELA cells (Additional file 1: Figure S3h), suggesting that the combined action of several SWI/SNF members might be necessary to control the ES–ELA cell transition.

### RISC inactivation de-represses the translation of selected chromatin regulators and destabilizes pluripotency

**Fig. 6:**
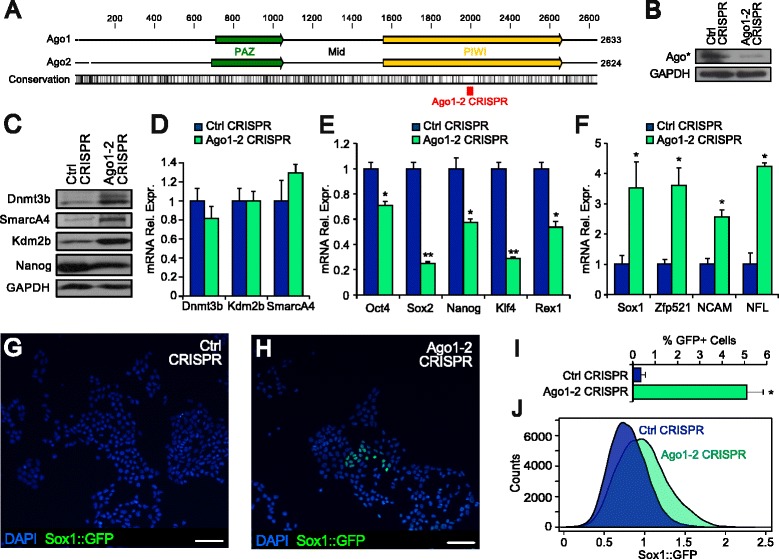
**a** CRISPR/Cas9 guide design: the single guide RNA (sgRNA; *red square*) was chosen to target a genomic region which is conserved between Ago1 and Ago2 but avoiding off-targets. **b** Western blot of Ago* proteins in control (*Ctrl*) or Ago1–2 CRISPR ES cells 4 days after transduction. **c** Western blot of Dnmt3b, SmarcA4, Kdm2b, Nanog, and GAPDH protein levels in ES cells cultured in 2i medium 4 days after transduction with Ago1–2 CRISPR lentiviral vector compared with control cells transduced with non-targeting CRISPR vector (*Ctrl CRISPR*). **d** mRNA levels of Dnmt3b, SmarcA4, and Kdm2b of cells as in **c**. **e**, **f** mRNA levels of pluripotency (**e**) or early neural commitment (**f**) in cells as in **c** and **d** 8 days after transduction. **g**, **h** GFP immunodetection in a 46C Sox1::GFP mESC line cultured in 2i medium 8 days after transduction with Ctrl CRISPR (**g**) or Ago1–2 CRISPR lentiviral vector (**h**). *Scale bars*, 50 microns. **i**, **j** Cell count (**i**) and cytofluorimetric analysis (**j**) of GFP-positive cells as in **g** and **h**. *p = 0.05, **p = 0.01, ***p = 0.001 (**e**–**f**, REST randomization test; **i**, Student’s *t*-test). Error bars show standard error

**Fig. 7:**
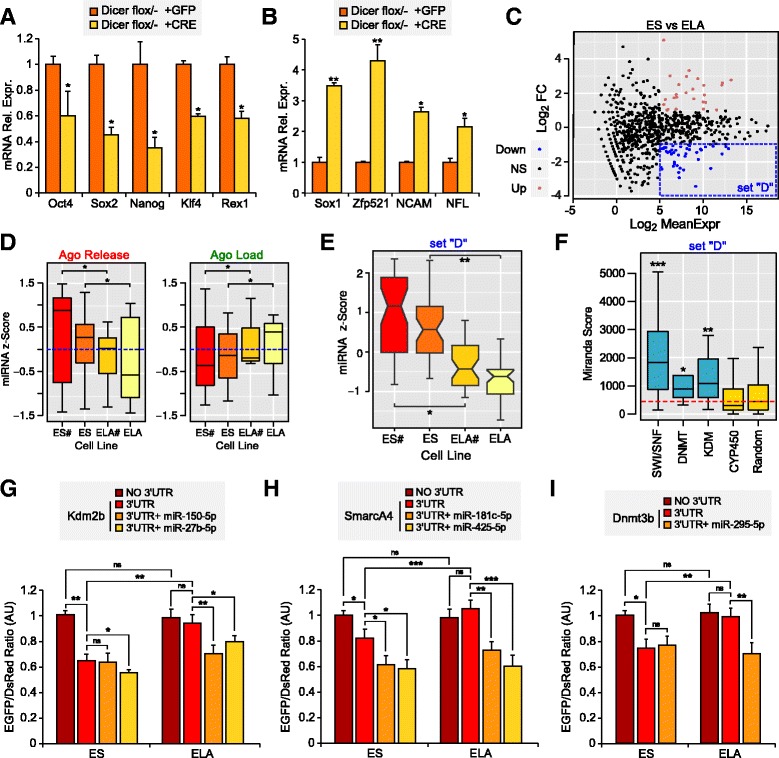
**a**, **b** RT-PCR analysis of mRNA levels of pluripotency (**a**) or early neural commitment (**b**) markers in Dicer flox/- conditional ES cells [33] 8 days after transduction with a CRE-GFP carrying lentiviral vector. Values are relative to β-actin mRNA expression. Expression levels were normalized to GFP-transduced cells. **c** Log_2_ fold change (*FC*; *y-axis*) and mean value of miRNA expression (*x-axis*) between ES and ELA cells. The *blue dashed box* indicates miRNAs significantly down-regulated (set *“D”*; mean log_2_ RPM ≥ 5 and log_2_ fold change ≤ −1) during priming. **d** Z-scores of the expression of miRNAs predicted to bind top Ago-released (ΔE < −2; *left panel*) or top Ago-loaded (ΔE > 2; *right panel*) mRNAs during the ES–ELA transition. Global miRNA/mRNA binding prediction was performed by the miRVestigator framework, which is designed to take as input a list of co-expressed genes and return the miRNA most likely regulating these genes [82]. miRNA expression was evaluated by small RNA-seq of ES cells cultured in 2i medium and LIF (*ES#*), ES cells cultured in LIF/serum (*ES*), ELA cells obtained from ES in 2iL (*ELA#*), and ELA obtained from ES. Ago enrichment variation provides a good estimation of miRNAs changing at the ES–ELA transition. **e** Z-scores of expression (RPM) of miRNAs selected in the *blue box* in **c** (set “D”), in ES cells cultured in 2i medium and LIF (*ES#*), ES cells cultured in LIF/serum (*ES*), ELA cells obtained from ES in 2iL (*ELA#*), and ELA obtained from ES (*ELA*). **f** The distribution of predicted binding affinity, calculated as cumulative Miranda scores of set “D” miRNAs in the 3′ untranslated region (UTR) of genes from the indicated families. The *dashed red line* marks the median score of a random gene set. **g**–**i** Normalized enhanced GFP (EGFP)/DsRed fluorescence ratios as obtained by co-transfection of plasmids and miRNA mimics/controls in ES and ELA cells (see “Methods”). All values, normalized on a DsRed plasmid as an internal control for transfection efficiency, are relative to the ratio displayed by ES cells transfected with an EGFP plasmid devoid of any 3′ UTR. *AU* arbitrary units.*p = 0.05, **p = 0.01, ***p = 0.001; **a**, **b**, REST randomization test; **d**–**f**, Wilcoxon test between pairs of conditions (**d**, **e**) or between each family and a randomized set of 3′ UTRs (**f**; see “Methods”); **g**–**i**, Student’s *t*-test

In order to evaluate the impact of global miRNA activity in ES cells, we performed acute RISC functional inactivation. We used a CRISPR/Cas9 lentivector (see “Methods”) with an RNA guide targeting both Ago1 and 2 (Fig. 6a, b; Additional file 1: Figure S4b), which are the only Argonaute genes expressed in ES cells (Additional file 1: Figure S4a). RISC down-regulation resulted in a dramatic increase in Dnmt3b, SmarcA4, and Kdm2b protein levels (Fig. 6c) without significantly affecting their mRNA (Fig. 6d). This increase in protein level was also observed when the mTOR pathway or the protein degradation pathway were pharmacologically inhibited, although to a lower extent due to the general decrease/increase in protein levels (Additional file 1: Figure S4c). This indicates that RISC-mediated control of these proteins occurs and is independent from their rate of translation/degradation. In addition, RISC inactivation in ES cells cultured in 2i medium caused down-regulation of pluripotency markers and up-regulation of early neural markers, including Zfp521, which is essential and sufficient for driving the intrinsic neural differentiation of mESC [32] (Fig. 6e–j). Down-regulation of pluripotency markers occurred also in ES cells cultured in the presence of LIF, either in 2i medium or in serum-containing ES cell medium (not shown). Similar results were obtained using a conditional Dicer flox/- cell line [33] upon Dicer excision with a CRE-carrying lentiviral vector (Fig. 7a, b). These data indicate that acute global miRNA down-regulation results in pluripotency destabilization.

### Ground-state miRNAs affect the levels of chromatin regulators

To get a better insight into the mechanisms of RISC targeting, we performed small RNA-seq of cells during the ES–ELA transition. This experiment had the additional advantage of validating the robustness of Ago-RIP as a method to measure RISC-mediated inhibition of mRNAs. Indeed, we observed that miRNAs predicted to target the most enriched or depleted RNAs in the Ago-RIP were accordingly up- or down-regulated during the ES–ELA transition (Fig. 7c, d). This result suggests that our method of analysis of mRNA/Ago enrichment is indeed predictive of miRNA–mRNA interactions.

More interestingly, the analysis of global miRNA expression at the ES–ELA cell transition highlighted a number of up- and down-regulated miRNAs (Additional file 1: Figure S4d; Additional file 5: Table S5), consistent with the differential pattern of expression observed in naïve and primed pluripotent cells [34, 35]. Specifically, the level of expression of these miRNAs is higher in naïve pluripotent cells and lower in ELA generated from ES cells (Fig. 7e), making them part of a signature of ground-state pluripotency. We found that the 3′ untranslated regions (UTRs) of genes coding for DNMT, KDM, and SWI/SNF are significantly enriched in predicted binding sites for the miRNAs down-regulated at the ES–ELA cell transition compared with control 3′ UTRs (Fig. 7f). High-affinity interactions predicted between 43 miRNAs most changing at the ES–ELA cell transition and distinct members of DNMT, KDM, and SWI/SNF are reported in Additional file 5: Table S5. Altogether, our observations suggest a direct role of miRNAs in inhibiting the translation of DNMT, KDM, and SWI/SNF in ES cells.

Since the high number of predicted miRNA–mRNA interactions impeded their comprehensive experimental validation, we focused on the effects of the miRNAs showing the strongest predicted interaction with Kdm2b (mmu-miR-150-5p and mmu-miR-27b-5p), SmarcA4 (mmu-miR-181c-5p and mmu-miR-425-5p), and Dnmt3b (mmu-miR-295-5p) (Additional file 5: Table S5). To evaluate miRNA–mRNA interactions, we co-transfected mature miRNAs together with reporters carrying enhanced GFP (EGFP) followed by the 3′ UTR of Kdm2b, SmarcA4 or Dnmt3b in ES cells. We then indirectly evaluated the amount of EGFP produced after 48 h in ELA cells or in ES cells maintained in ES cell medium, measuring the ratio between EGFP fluorescence and the fluorescence produced by a DsRed-carrying plasmid (Fig. 7g–i; see “Methods”). EGFP of 3′ UTR-carrying vectors was always expressed at lower levels than EGFP of empty vector. In the presence of Kdm2b, SmarcA4, or Dnmt3b 3′ UTRs, however, EGFP levels were significantly lower in ES cells than in ELA cells, indicating that a translational switch driven by these 3′ UTRs occurs at the ES–ELA transition. Moreover, all the miRNAs expected to bind to the 3′ UTRs with the best efficiency were able to inhibit EGFP expression from a 3′ UTR-carrying vector in ELA cells. According to the prediction of multiple miRNAs interacting with one 3′ UTR, the co-transfection of one single miRNA was not able to inhibit EGFP expression to the levels observed in ES cells. Finally, EGFP expression was less inhibited by miRNA co-transfection in cells maintained in ES medium (ES cells) than in cells differentiated in chemically defined minimal medium (CDMM; ELA cells), in agreement with the evidence that higher amounts of these miRNAs are already present in ES cells and, therefore, their transfection is expected to show a smaller additional effect. These results support the specificity of some of the mRNA–miRNA interactions at the ES–ELA cell transition predicted by in silico tools.

## Discussion

Taking advantage of the spontaneous differentiation program that ES cells undergo when deprived of pluripotency-maintaining signals, we isolated and studied cells primed to differentiation (ELA) just after their escape from the ground-state pluripotency state.

We performed Ago-RIP as a way to estimate RISC-mediated inhibition of gene expression. Two new nuclear roles for Ago2 have been recently found in addition to its main function as a component of RISC: one in pre-mRNA splicing and one in transcriptional repression [36]. We tend to exclude an influence of nuclear RNA in our analysis as Ago–RNA immunoprecipitation was performed on cytosolic protein fractions and strong depletion of nuclear RNA species in Ago–RNA was confirmed by the analysis of gene expression arrays (not shown). Moreover, in light of the strong anti-correlation observed when comparing Ago enrichment with ribosome enrichment (Fig. 4a) and the high consistency between Ago-enriched mRNAs and their predicted targeting miRNAs at the ES–ELA cell transition, we think that the ratio of RNA splice junctions possibly immunoprecipitated by Ago was negligible.

The strong correlation between the decrease of Ago–RNA and the increase of ribosome occupancy and MS detection at the ES–ELA transition strongly supports a protein translation control. However, the protein changes as measured by MS were bigger than ribosome occupancy. We should consider that the normalization method applied for polysome profiling did not take into account the general up-regulation of the translational machinery occurring during cell priming but only measured the modulation of ribosome occupancy above (or below) the overall change (see “Methods”). This, together with the fold change in ribosome occupancy exceeding the level of total RNA up-regulation, would account for the discrepancy.

We observed that the strongest variation, in terms of the number of mRNAs changing their association with Ago, happened at the ES–ELA transition, suggesting an important role for this mechanism of protein translation control in the maintenance of ground-state pluripotency.

A number of mRNAs released by Ago are related to DNA repair and replication. As ES cells and ELA correspond to inner cell mass (ICM) and post-implantation cells, respectively, the requirement of increased proliferation during priming is consistent with the higher cell division rate occurring after embryo implantation [21].

Chromatin regulators, whose role in pluripotency has been extensively discussed [2], are the other main class of mRNAs released by Ago at the ES–ELA transition. We found that DNMT, KDM, and SWI/SNF mRNAs are already present in ground-state pluripotent cells but their translation is either inhibited or kept at lower levels.

During early embryonic development, DNMT activity contributes to establish different genomic methylation patterns, which characterize somatic differentiated cells [37]. The observations that a number of pluripotency-related genes are hypomethylated in stem cells [38] and that global DNA hypermethylation occurs in epiblast cells [39] suggest that the extensive reprogramming of genomic methylation pattern is a key event in ES cell priming. Global DNA hypomethylation is associated with ground-state pluripotency [40–43] but, surprisingly, appreciable levels of DNMTs are transcribed in naïve ES cells [44]. Our finding that Dnmt1/3 mRNAs are significantly released by RISC and that Dnmt1/3 protein translation is enhanced at the ES–ELA transition might contribute to explain the presence of DNMT mRNAs in ESCs.

Members of the KDM and SWI/SNF families that we found to be released from Ago at the ES–ELA transition can also act as transcriptional repressors. Kdm2b (Jhdm1b) is responsible for the demethylation of H3K36 [45], which marks the transcribed region of active genes [46], while SmarcA4 is part of a complex that is necessary to silence Nanog upon differentiation [31]. Indeed, inhibiting the increase of SmarcA4 impedes the ES–ELA transition when LIF and serum are removed from the culture medium. While this fact is not in contrast with the requirement of SmarcA4 for the maintenance of pluripotency [47], it confirms and accounts for the evidence that SmarcA4 activity is necessary for ES neuralization [4]. Overall, despite the fact that these chromatin regulators are important for the maintenance of pluripotency, we found that their up-regulation is crucial for the control of priming.

Although we do not exclude that transcriptional regulation of DNMT, KDM, and SWI/SNF might contribute to cell priming, our results suggest that the release of selected chromatin regulators from RISC inhibition during priming may play a central role in reducing the pervasive transcription observed in ES cells [4] by reshaping the nuclear landscape of the cell [22]. Moreover, the release from RISC inhibition of DNMT, KDM, and SWI/SNF members could explain the intrinsic tendency of ES cells to begin differentiating in the absence of external signals [48].

Naïve and primed mESC have distinct miRNA signatures, with only a few genomic clusters accounting for the majority of the difference in miRNA expression profiles [34, 35]. Global miRNA expression analysis of our in vitro model revealed a consistent modulation during the transition from ES to ELA cells. In agreement with the literature [34, 35], members of the miR-302/367 cluster, which are more expressed in EpiSC, and the miR-290/295 cluster, which are more abundant in ES cells, were up- and down-regulated, respectively, at the ES–ELA transition. The role of miRNAs in ES cell cycle regulation, self-renewal, and their ability to differentiate has been extensively assayed using cell lines in which key components of miRNA processing, Dicer or Dgcr8, were inactivated [49–51]. Moreover, recent studies of global gene expression profiling performed at the single-cell level highlighted that Dgcr8-deficient ES cells are more similar to ES cells cultured in 2i and LIF rather than in serum and LIF [52] and thus suggested that miRNAs act as key mediators of the transition from ground state pluripotency to primed states, with their absence mimicking the inhibition of the Erk and GSK3 signaling pathways observed in 2i culture. This observation, together with many in vitro studies on the impact of miRNAs in pluripotency, suggest that miRNAs are dispensable for maintaining a ground state of pluripotency in vitro [52]. However, Dicer inactivation dramatically down-regulated Oct4 in the epiblast, causing the loss of stem cells, possibly due to their premature differentiation [7]. In our work, we tried to solve this apparent contradiction between the in vitro and in vivo results by using transient Dicer inactivation assays in vitro. To the best of our knowledge, a key issue of all the in vitro studies of Dicer and Dgcr8 inactivation published so far is that they were performed in cell lines obtained through extensive cell culture selection and stabilization. For this reason, their use neither permitted the role of gene expression buffering exerted by miRNAs in naïve pluripotent cells to be studied nor allowed analysis of the changes that are “naturally” occurring during cell priming. Indeed, by acutely inactivating Dicer or RISC, we found that global miRNA activity is required to maintain pluripotency and that its perturbation triggers the beginning of a differentiation program in ES cell cultures which corresponds to what is observed in vivo.

Our observations do not allow us to evince the exact set of miRNAs that are necessary to maintain pluripotency through the protein translation control of chromatin regulators. By in silico analysis, we identified quite a large number of miRNAs whose modulation at the ES–ELA transition and predicted binding to DNMT, KDM, and SWI/SNF mRNAs might support a direct role in cell priming. For five of these, we showed the ability to inhibit protein translation through the predicted 3′ UTR target. Nevertheless, the high number of DNMT, KDM, and SWI/SNF members possibly involved in the control of cell priming and the consequent abundance of miRNAs predicted to be interactors suggest that a large set of miRNAs might be involved in orchestrating the inhibitory control of priming. The precise characterization of each single miRNA–mRNA interaction is beyond the scope of this work.

## Conclusions

**Fig. 8:**
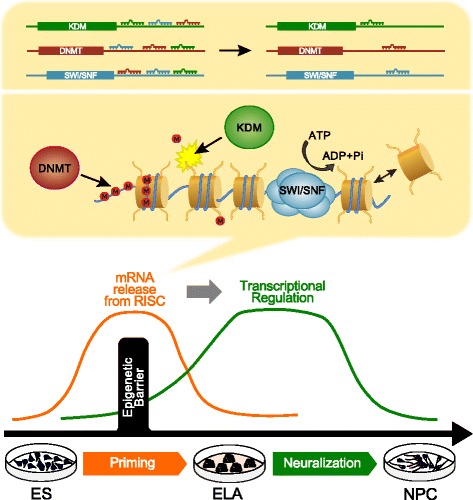
Two-layer model of epigenetic control of pluripotency. A marked release from RISC characterizes the ES–ELA transition (priming), whereas transcriptional regulation occurs preferentially at the ELA–NPC transition (neuralization). Our data suggest a causal link between the two phenomena as the pool of de-repressed genes contains distinct chromatin regulators which could overcome the epigenetic barrier between the primed state and ground-state pluripotency. Thus, ground-state miRNAs, which are down-regulated during priming, could shield naïve pluripotent cells from the epigenetic transition required for the onset of embryonic differentiation programs

ES cell self-renewal is controlled by a small gene regulatory circuitry comprising a dozen interacting key transcription factors whose activity seems sufficient to maintain ground state pluripotency [53]. Nevertheless, players other than transcription factors are likely to control the choice between self-renewal and differentiation of ES cells. For instance, a global increase of protein translation enforced by a hierarchy of translational regulators is a feature of the switch between stem cell self-renewal and differentiation [54]. This observation, together with the evidence of pervasive transcription occurring in ES cells [4], supports the hypothesis that a tight control of protein translation, in addition to transcriptional regulation, might account for ground state pluripotency. This is consistent also with the observation that RNA interference impairment in vivo causes the loss of stem cells [7]. Our findings suggest that the phenotype obtained in vivo after Dicer inactivation could be caused by disregulated translation of key chromatin regulators and place RISC-mediated control of protein translation among the general mechanisms accounting for the control of cell pluripotency. If this suggests a primary mechanism of miRNAs in preserving ES cell pluripotency and inhibiting the onset of embryonic differentiation programs, on the other hand miRNA-mediated control of chromatin regulators might maintain cells in a metastable state, which could rapidly be converted into priming to cell differentiation upon the removal of stemness-sustaining factors. Accordingly, priming could be seen as a process in which two epigenetic mechanisms are layered one on the other (Fig. 8): a first layer, which is microRNA-mediated, on which lies a second layer consisting of chromatin-based modulation of transcription.

## Methods

### ES culture and neuralization

Murine ES cell lines E14Tg2A, 46C, and TNG-A (transgenic Sox1-GFP and Nanog-GFP ES cells, kindly gifted by Dr. A. Smith, University of Cambridge, UK) [16, 48] and the Dicer flox/null ES cell line (generously provided by Dr. G. Hannon, Cancer Research UK Cambridge Institute) [33] were cultured on gelatin-coated tissue culture dishes at a density of 40,000 cells/cm^2^. ES cell medium, which was changed daily, contained GMEM (Sigma), 10 % fetal calf serum, 2 mM glutamine, 1 mM sodium pyruvate, 1 mM non-essential amino acids, 0.05 mM β-mercaptoethanol, 100 U/mL penicillin/streptomycin, and 1000 U/mL recombinant mouse LIF (Invitrogen). For ground-state reprogramming of ES cells, medium was switched for three to five passages to 2iL medium: GMEM (Sigma) supplemented with N2/B27 (no vitamin A; Invitrogen), 2 mM glutamine, 1 mM sodium pyruvate, 1 mM non-essential amino acids, 0.05 mM β-mercaptoethanol, 100 U/mL penicillin/streptomycin, 1 μM MEK inhibitor PD0325901, 3 μM GSK3 inhibitor CHIR99021, and 1000 U/mL recombinant mouse LIF [55].

Chemically defined minimal medium (CDMM) for neural induction consisted of DMEM/F12 (Invitrogen), 2 mM glutamine, 1 mM sodium pyruvate, 0.1 mM non-essential amino acids, 0.05 mM β-mercaptoethanol, and 100 U/mL penicillin/streptomycin supplemented with N2/B27 (no vitamin A; Invitrogen). The protocol of ES neuralization consisted of three steps (Fig. 1a). During step 1, dissociated ES cells were washed with DMEM/F12, aggregated in agar-coated culture dishes (65,000 cells per cm^2^), and cultured as floating aggregates in CDMM for 2 days. The second day, 75 % of the CDMM was renewed.

In step 2, ES cell aggregates were dissociated and cultured in adhesion (65,000 cells per cm^2^) on poly-ornithine (Sigma; 20 μg/ml in sterile water, 24 h coating at 37 °C) and natural mouse laminin (Invitrogen; 2.5 μg/ml in PBS, 24-h coating at 37 °C) for 4 days, changing CDMM daily. In step 3, after a second dissociation, ES cells were cultured for an additional 4 days in CDMM devoid of B27 supplement to drive terminal differentiation, using the same type of seeding density and coated surface described for step 2. Serum employed for trypsin inactivation was carefully removed by washing with DMEM/F12. LIF serum withdrawal coupled to culturing as cell aggregates for the first 2 days results in a very low amount of cell death [9]. Cell viability, which was monitored by a trypan blue exclusion test and cell counting, was more than 90 % and did not vary significantly between different steps of the differentiation protocol. Analysis of global gene expression on GO terms of apoptosis between ES and ELA cells showed no significant change (GO 0006917, induction of apoptosis, *p* = 0.668; GO 1900117, regulation of execution phase of apoptosis, *p* = 0.373; GO 0097194, execution phase of apoptosis, *p* = 0.559). The following factors were tested by addition during step 1: 5-azacytidine (Sigma-Aldrich; 50 nM) and 5-carboxy-8-hydroxyquinoline (Sigma-Aldrich; 100 μM).

### Ago-bound RNA immunoprecipitation

In order to isolate cellular mRNAs that are bound by Ago proteins, we used a RIP-Assay Kit for microRNA (MBL Japan) according to the manufacturer’s instructions. The protocol includes a step for the isolation of the cytosolic fraction, which allowed us to avoid the co-precipitation of nuclear Ago and RNAs. Anti-pan Ago antibody, clone 2A8 (Ago*; Millipore MABE56) [56], mouse total IgG (Millipore; 12-371), or Anti-Ago2 antibody, clone 9E8.2 (Millipore; 04-642) were used for immunoprecipitation (IP). For each single experiment, we started from 10^7^ cells collected by pooling three (ES) to ten (ELA, NPC, NPC/NEU Neu) different samples. The binding of RNP to the beads was confirmed after IP by western blotting with anti-Ago* antibody (Additional file 1: Figure S1a). The global screening of Ago–RNA by microarray analysis was performed following IP with Anti-pan Ago antibody (see the next section). Three independent experiments were performed. IP with Anti-Ago2 antibody was used to validate association of selected mRNAs with Ago by RT-PCR using a spike-in as a reference (Additional file 1: Figure S2f, g). Tol2 transposase mRNA (8 ng) synthesized in vitro was added to each pellet as a spike-in before IP. Mouse total IgGs were always used as an IP negative control. Two independent experiments were performed.

### Microarray hybridization and data analysis

Total RNA was extracted with NucleoSpin RNA II columns (Macherey-Nagel). RNA quality was assessed with an Agilent Bioanalyzer RNA 6000 Nano kit; 200 ng of RNA was labeled with Low Input Quick Amp Labeling Kit, One-Color (Agilent Technologies, purified and hybridized overnight onto an Agilent SurePrint G3 Mouse Gene Expression Array (8x60K) before detection according to the manufacturer’s instructions. An Agilent DNA microarray scanner (model G2505C) was used for slide acquisition and spot analysis was performed with Feature Extraction software (Agilent Technologies).

Data were background-corrected and quantile normalized among arrays using the Bioconductor package *limma* [57]. In order to sort out Ago-released or Ago-loaded mRNAs in Fig. 1c, we employed the following analysis pipeline (schematized in Additional file 1: Figure S1b):

1. For a gene probe to be considered significantly bound to Ago, we assumed that the distribution of signal is bimodal, with a fraction of genes not bound (first peak, background) and another displaying a heterogeneous degree of binding. In fact this is what we observed in the distribution of fluorescence intensity (Additional file 1: Figure S1c). After fitting the distribution to a Gaussian mixture model [58], we set a threshold T_1_ equal to (μ_1_ + 3σ_1_), which corresponds to *p* = 0.001 of false positive bound mRNA. Only mRNAs above this threshold were taken into account.
2. For each step, Ago enrichment (E_n_) was evaluated as log_2_ ratio between Ago–RNA and total RNA levels after quantile normalization and an arbitrary threshold of T_2_ ≥ 2 was applied in order to capture only the most enriched genes. Conversely, genes with enrichment less than 0.5 were considered not significantly enriched (T_3_; this value was relaxed to 1.2 for subsequent analyses; e.g., GO analysis and Fig. 4b–d).
3. For each transition, genes released from Ago have E_n_ ≥ T_2_ and E_n+1_ ≤ T_3_ (as depicted in Additional file 1: Figure S1b, right panel) while, conversely, genes loaded by Ago have E_n_ ≤ T_3_ and E_n+1_ ≥ T_2_.
4. Finally, a threshold on the differential enrichment between steps (ΔE = E_n+1_ − E_n_) was used as a filter to sort out top regulated genes (|ΔE| ≥ 1.5).

Gene ontologies over-represented in the subset of Ago-released genes during the ES–ELA transition were evaluated using the web service Database for Annotation, Visualization and Integrated Discovery (DAVID) [59].

Genes that were differentially expressed between ES and ELA cells with |log_2_FC| ≥1 (where FC is fold change), corrected *p* value <0.05, and mean log_2_ expression ≥7.5 (n = 1207; Additional file 6: Table S4) were used for hierarchical clustering in order to assess the effect of manipulations. RNA from three different sets of treatments was pooled.

We changed the cutoffs for different analyses of global mRNA expression because the dynamic ranges of mRNA change were different in the different experimental settings: we used log_2_ > 2.5 when comparing ES, ELA, NPC, and neuron cells, which are very different from each other, while we used log_2_ > 1 when comparing ES cells with ELA cells, which are more similar.

Euclidean distance and a complete linkage algorithm were employed for clustering.

To assay the concordance of gene modulation between different conditions and obtain the statistical significance, we used a sign test. The sign test was performed comparing the fold change of ES–ELA cells to the fold change of treated/control ELA cells for each gene. Randomized sets of data used as control were obtained by substituting the fold changes of treated/control datasets with fold changes of random genes (randomization 1) or by scrambling the order of treated/control ELA cell values (randomization 2).

### Profiling of ribosome-associated RNA (polysome profiling)

For the pull-down of ribosome-associated RNA we were inspired by the TRAP (translating ribosome affinity purification) protocol developed in the laboratory of Dr. Nathaniel Heintz [26, 27]. In short, this protocol is based on the pull-down of ribosomes using the large subunit protein Rpl10a, which is fused with GFP. We modified this protocol in order to pull down the endogenous Rpl10a protein as this would allow us to sample translation without altering the endogenous translational machinery. Immediately before the analysis, ES and ELA cells were trypsinized and resuspended in the appropriate growth medium supplemented with 100 μg/mL cycloheximide in order to stall ribosomes. After a 15 min incubation at 37 °C, cells were recovered and washed with ice-cold PBS plus 100 μg/mL cycloheximide to remove traces of serum and medium. Each sample (for both ELA and ES cells) included approximately 20 million cells and we conducted all experiments in duplicate. The samples were resuspended in 1 mL ice-cold lysis buffer (20 mM HEPES/KOH pH 7.3, 150 mM KCl, 10 mM MgCl_2_, 1 % IGEPAL CA 630 (Sigma), 0.5 mM DTT, 100 μg/mL cycloheximide, 1 pill/10 mL complete MINI EDTA free protease inhibitor (Roche), 3 μL/mL RNAsin RNAse inhibitor (Promega), 3 μL/mL SUPERASEin RNAse inhibitor (Life Techologies)) and incubated on ice for 10 min before being triturated in a chilled dounce homogenizer with approximately 20 strokes. After lysis was completed, cellular debris (mostly formed by nuclei and fragments of cell membrane) was removed by centrifugation at 2000 RCF for 10 min. After centrifugation, 0.1 volumes of 300 mM 1,2-diheptanoyl-sn-glycero-3-phosphocholine (DHPC, Generon Ltd) were added to each sample to further solubilize ribosomes, followed by a 10 min incubation on ice and by a second centrifugation at 20,000 RCF for 10 min. The final clarified lysate was transferred to a new low-retention tube and 0.1 volumes were removed to be used as “input” samples for the measurements of total RNA levels. To immunoprecipitate ribosomes, 16 μg of anti-Rpl10a antibody (Sigma, catalogue number WH0004736M1) or an equivalent amount of anti-GFP antibody (control, Sigma catalogue number G1544) were added to each sample and incubated overnight with end-to-end agitation at 4 °C. The following day, 200 μL of 30 mg/mL Protein-G Dynabeads (Life Technologies) were washed in PBS plus 0.1 % Tween 20 (PBST), blocked in PBST plus 1 % bovine serum albumin (BSA) for 1 h, and added to the lysate/antibody mix. Beads were incubated overnight at 4 °C with end-to-end agitation. The following day the precipitated ribosomes were washed four times in 1 mL high-salt buffer (20 mM HEPES/KOH pH 7.3, 350 mM KCl, 10 mM MgCl_2_, 1 % IGEPAL CA 630 (Sigma), 0.5 mM DTT, 100 μg/mL cycloheximide) and RNA was extracted using the RNEasy Micro kit (Qiagen) as per the supplier’s protocol. The results were quantified by spectrophotometry (Nanodrop) and capillary electrophoresis (Bioanalyzer RNA Nano kit; Additional file 1: Figure S2b). RNA (2 μg) was used to prepare each RNA-seq library. Purified RNA was ribosome-depleted using the RIBO-Zero Gold kit (Illumina), which uses magnetic beads conjugated to rRNA-specific oligonucleotides to remove ribosomal RNA. The final purified RNA was analyzed by capillary electrophoresis (Bioanalyzer Pico Kit) and retro-transcribed and cloned into a sequencing library using the SCRIPTSeq v2 library preparation kit (Illumina) according to the supplier’s protocol. Sequencing libraries were evaluated by Bioanalyzer (to verify the size distribution of fragments) and quantified by quantitative PCR using primers specific for Illumina adaptors (KAPA library quantification kit). All eight libraries (two replicates for ELA and ES cells, both IP and input) were mixed at a similar final concentration before cluster generation and sequencing on a HiSeq 2500 instrument.

### Polysome profiling data analysis

All the libraries were sequenced in a multiplexed lane using v4 chemistry on a Illumina HiSeq instrument, producing approximately 250 million reads of raw data. After demultiplexing, each library comprised between 20 and 50 million reads, with the input library overall producing fewer reads than the ribosome IP libraries. This effect was most likely due to an unequal loading and to the different nature of the input RNA samples (including a variety of RNA and small RNA types, while the ribosome IP libraries were mostly composed of messenger RNAs) and raised no concern for the subsequent analysis (Additional file 1: Figure S2a).

All the datasets were subject to quality control using the fastx toolkit software through the following analyses: (1) quality score per cycle, (2) nucleotide distribution per cycle, (3) PCR duplicates (estimated by collapsing the reads). All libraries yielded good quality data, with the exception of one of the replicates for the ELA input sample, which showed a lower complexity and higher amount of duplication (resulting in lower mapping to the transcriptome during the later steps of the analysis; data not shown).

Reads contained in each of the remaining libraries were mapped to the mouse transcriptome (USCS mm10 release) using the software TopHat2 [60], which accounts for spliced transcripts. Repeated regions (i.e., transposable elements) were excluded and a maximum of two hits on the genome and two mismatches were allowed during mapping. Mapped reads were assigned to genes using the htseq-count script [61], which counts features overlapping each gene in the released transcriptome. Counts were calculated for each *gene_id*, collapsing eventual splicing isoforms. The resulting raw counts were normalized using the DESeq2 R package [62]. The DESeq scaling factor for a given lane is computed as the median of the ratio, for each gene, of its read count over its geometric mean across all samples. It is important to notice that this normalization (and, effectively, any other normalization applicable to our data) is based on the assumption that the majority of genes are not differentially expressed among samples. This is not necessarily true during differentiation as there are reports indicating a massive up-regulation of the translational machinery [54], which would imply a higher read number for most genes during the ES–ELA transition. Unfortunately, it is impossible for any “internal” normalization method to account for global changes. As a consequence, our analysis of differential translation only indicates as significant genes that vary above or below the overall change level. We believe that this further increases the significance of our findings since our results include genes subject to a specific regulation rather than a global effect.

After normalization, we analyzed our results to assess correlation among datasets and among conditions (through hierarchical clustering and principal component analysis; Additional file 1: Figure S2c, d). The results indicated that one of the replicates for the ELA input sample showed poor correlation with both the other input replicate and the other datasets (ELA.input.1; Additional file 1: Figure S2c, d). This, together with the lower mappability, lower read quality, and increased amount of PCR duplicates (as described above), led us to exclude this replicate from further processing. While this reduces the statistical power of our analysis, the fact that we are filtering our results to include only genes presenting high expression and a high degree of change in their ribosome enrichment makes us confident that our conclusions are still significant.

The remaining libraries were filtered to include only genes with an average of 50 counts in at least one of the two conditions (ES or ELA). This threshold was chosen to exclude less-expressed genes, which would be the most affected by the inherent noise of our method. For all the genes passing this first filter, ribosome enrichment was calculated by dividing the average ribosome IP count by the input count. A second filter was then applied, excluding genes for which the standard deviation of the counts accounted for more than 20 % of the average value. This filtering produced a list of approximately 5000 genes, for which we calculated a log_2_ fold change in ribosome enrichment (ELA average enrichment/ES average enrichment; log_2_RiboΔE). For our GO enrichment analysis, we considered only genes with log_2_RiboΔE >1 during differentiation, producing a final list of ~400 differentially regulated genes.

While the GO category enrichment results we obtained were all highly significant according to multiple statistics tests, we couldn’t properly assess the significance of changes in the ribosome enrichment of individual genes due to our decision to remove the faulty replicate in the ELA input dataset. We nevertheless filtered our data to exclude genes with high replicate-to-replicate variation and low expression and reported those with the highest changes, in both directions, in our raw data (Additional file 2: Table S1). Some of the genes individuated by our other analyses (Ago IP and protein MS) fell short of matching our filter in the ribosome enrichment data; nevertheless, they showed a clear increase in enrichment, which was consistent with the modulation highlighted by the other techniques. We decided to show these results since we believe that the fact that we obtained similar results through multiple and complementary analysis types constitute a strong proof of significance.

### Semiquantitative real-time PCR

Total RNA was extracted from ES cells or tissue samples with NucleoSpin RNA II columns (Macherey-Nagel). ES cells from at least two to three different wells of 24-well plates were always pooled together to compensate for variability in cell seeding. RNA quantity and RNA quality were assessed by gel electrophoresis. For each sample, 200 ng of total RNA was reverse-transcribed (Eurogentech, RT Core kit). Amplified cDNA was quantified using GoTaq qPCR Master Mix (Promega) on a Rotor-Gene 6000 (Corbett). Primers used for amplification which were not previously reported [9] were taken from PrimerBank [63]. Amplification take-off values were extracted using the built-in Rotor-Gene 6000 relative quantitation analysis function and relative expression was calculated with the 2^-ΔΔCt^ method, normalizing to the housekeeping gene β-actin. Standard errors shown as error bars in all histograms were obtained from the error propagation formula as described in [64]; the statistical significance of three independent experiments was probed with a randomization test using the REST software [65].

### Immunocytodetection

Cells prepared for immunocytodetection experiments were cultured on poly-ornithine/laminin-coated round glass coverslips. Cells were fixed using 2 % paraformaldehyde for 10–15 min, washed twice with PBS, permeabilized using 0.1 % Triton X100 in PBS, and blocked using 0.5 % BSA in PBS. Primary antibodies used for microscopy included Oct3/4 (1:200; Santa Cruz Biotechnology C-10; sc-5279), Nanog (1:300; Novus Biologicals; NB100-58842), acetylated N-tubulin (clone 6-11B-1; 1:500; Sigma; T7451), neuronal class III β-tubulin (1:500; Covance; MRB-435P), Nestin (1:200; Millipore; MAB353), Pax6 (1:400; Covance; PRB-278P), and GFP (1:1000; Life Technologies; A-6455). Primary antibodies were incubated for 2 h at room temperature; cells were then washed three times with PBS (10 min each). Alexa Fluor 488 and Alexa Fluor 568 anti-mouse or anti-rabbit IgG conjugates (1:500; Molecular Probes; A-11001 and A-11011, respectively) were incubated for 1 h at room temperature in PBS containing 0.5 % BSA for primary antibody detection, followed by three PBS washes (10 min each). Nuclear staining was done with DAPI (2 μg/mL; Sigma).

The same protocol was applied on sections of embryoid bodies after fixation (2 h at 4 °C in 2 % PFA), dehydration (overnight at 4 °C in 30 % sucrose/PBS), inclusion in Tissue-Tek OCT compound (Sakura), and cryostat sectioning (12–16 μm) on SuperFrost glass slides (Thermo).

### Nuclear extract preparation and proteomics sample pre-processing

About 10^7^ cells per sample were dissociated, washed with ice-cold PBS, and resuspended in 400 μL of nuclear extraction buffer (50 mM Tris-Hcl pH 7.9, 10 mM KCl, 0.2 % NP40, 10 % glycerol, and 1 mM PMSF) for 3 min in ice, then nuclei were pelleted by centrifugation at 6000 RPM for 3 min at 4 °C. Nuclei were washed twice with 1 mL of nuclear extraction buffer devoid of NP40; before proceeding to mass spectrometry analysis, a small aliquot was lysed in loading buffer and used for western blotting to confirm that the enrichment in nuclear fraction was homogeneous between samples.

Nuclear extracts were lysed using lysis buffer containing TRIS HCl 50 mM pH 8.1, 0.5 % Triton X-100, and 0.25 % deoxycholic Na and sonicated. Samples were centrifuged at 10,000 g for 10 min at 4 °C to discard cell debris and buffer detergent was removed using a detergent removal spin column (Pierce, Thermo Scientific, USA). Protein concentration was determined by bicinchoninic acid assay (Pierce, Thermo Scientific, USA). We diluted 100 μg of protein in 25 mM of ammonium hydrogen carbonate (pH 8) and reduction was obtained by adding 5 mM dithiothreitol with an incubation of 20 min at 80 °C. Iodoacetamide was added to the samples to a final concentration of 10 mM and incubated in the dark for 30 min at 37 °C. Digestion was performed incubating overnight with 100:1 substrate:trypsin (Roche, Germany) at 37 °C. Nucleic acid contaminants were removed with Amicon Ultra-3 K centrifugal devices (Merck Millipore, Germany), then flow-through containing peptides was loaded on a C18 cartridge in order to eliminate debris and filtered with 0.22 μm filter.

### Liquid chromatography-tandem MS analysis

Chromatographic separation of peptides was performed using a nano-HPLC system (Eksigent, ABSciex, USA). The loading pump pre-concentrated the sample in a pre-column cartridge (C18 PepMap-100, 5 μm 100 A, 0.1 x 20 mm; Thermo Scientific, USA).

Chromatographic separation of peptides was performed using a C18 PepMap-100 column (3 μm, 75 μm × 150 mm; Thermo Scientific, USA) at a flow rate of 300 nL/min. Runs were performed with eluent A (Ultrapure water, 0.1 % formic acid) under a 60 min linear gradient from 25 to 40 % of eluent B (acetonitrile (Romil, UK), 0.1 % formic acid) followed by 10 min of a purge step and a 20-min re-equilibration step. The column was directly coupled to a TripleTOF 5600 System (ABSciex, USA), equipped with a DuoSpray ion source (ABSciex, USA). Peptides eluted from chromatography were directly processed using a TripleTOF 5600 mass spectrometer (ABSciex, USA). The mass spectrometer was controlled by Analyst TF 1.6.1 software (ABSciex, USA). For information-dependent acquisition (IDA) analysis, survey scans were acquired in 250 ms and 25 product ion scans were collected if exceeding a threshold of 125 counts per second (counts/s). Dynamic exclusion was set for one-half of peak width (∼8 s), then the precursor was refreshed off the exclusion list. MS/MS data were processed with ProteinPilot Software (ABSciex, USA) using the Paragon and Pro Group Algorithms and UniProt 2013 as protein database for *Mus musculus*. The false discovery rate (FDR) analysis was done using the integrated tool in the ProteinPilot software and a confidence level of 95 % was set. Label-free statistical comparative analysis [66] was performed using MarkerView (ABSciex, USA). The ion chromatograms of high confidence peptides identified by ProteinPilot were extracted using PeakView Software (ABSciex, USA), then MS peak areas and identifications were imported into MarkerView. Normalization of the total plaque area (plaque size) was done using a global normalization of profiles (total protein content). Principal component analysis (data not shown) was performed in order to determine groupings among the data set. The two groups (ES and ELA, n = 3) were compared with *t*-test using a threshold *p* value ≤0.05 and fold change >2.

### Western blotting

Cells were lysed with RIPA buffer (50 mM Tris-HCl pH 7.6, 1 % NP40, 0.5 % deoxycholic acid, 150 mM NaCl, 1 mM EDTA, 1 mM PMSF, 1 % SDS) supplemented with Complete Protease Inhibitor Cocktail (Roche); lysate was incubated for 30 min on ice and sonicated three times for 10 sec each on medium power in order to reduce viscosity. Supernatant was harvested by centrifugation (10 min at 13,000 RPM, 4 °C) and quantified with a Micro BCA Protein Assay Kit (Thermo Scientific). Samples were denatured by adding sample buffer (LDS Sample Buffer, Thermo Scientific) and boiled for 10 min on a thermal block at 99 °C. The total protein extract (10 to 50 μg) was resolved on 8–10 % acrylamide gels, transferred on a nitrocellulose membrane (Hybond-c Extra, GE Healthcare), blocked with 5 % milk proteins in TBST (50 mM Tris pH 7.6, 150 mM NaCl, 0.05 % Tween-20), and probed with primary antibodies, including Dnmt3b (1:2000; Imgenex/Novus Biologicals; IMG-184A), SmarcA4 (1:1000; Abcam; ab4081), Kdm2b (1:2000; Merck Millipore; 09-864), PolII (clone CTD4H8; 1:1000; Millipore), α-tubulin (clone B-5-1-2; 1:5000; Sigma-Aldrich; T6074), GAPDH (Sigma-Aldrich; G9545), panAgo clone 2A8(1:250; Millipore; MABE56) and Ago2 clone 9E8.2 (1:2000; Millipore; 04-642). After 1 h of incubation, the membrane was washed three times with TBST (15 min each) and probed with a HRP-conjugated secondary anti-mouse or anti-rabbit antibody for 1 h (Santa Cruz Biotechnology; sc-2005 and sc-2030, respectively). After three more washes, signal was revealed by means of an enhanced chemiluminescence kit (G&E Healthcare) on a BioMax XAR Film (Kodak).

### Lentiviral vector construction and use

Knock-down vectors against SmarcA4 or luciferase (shSA4 and shCtrl, respectively) were constructed in order to sustain high expression of shRNAs in both ES and differentiating conditions: an IRES-PuroR-shRNA cassette from pGIPZ vectors (Open Biosystems) was excised by double restriction with NotI and MluI (NEB), filled in with T4 DNA polymerase (NEB), and inserted into a modified pWPXLd vector (Addgene number 12258; original GFP was swapped with DsRed with restriction sites MluI/XmaI) which was cut with NdeI and blunted as above. This resulted into a self-inactivating transfer plasmid carrying a DsRed-IRES-Puromycin resistance cassette with the shRNA of interest, driven by the strong ubiquitous promoter EF1α. As a reporter of human Nanog promoter activity we employed a PL-SIN-Nanog-EGFP vector [67] (Addgene, number 21321).

CRISPR/Cas9 vectors were based on LentiCRISPR v2 [68] (Addgene, number 52961) and constructed following the manufacturer’s instructions. Briefly, vector was linearized with BsmBI (NEB), dephosphorylated with calf intestinal phosphatase (NEB), and ligated with an oligo duplex obtained by annealing two single-stranded DNA sequences synthesized in vitro and 5′-phosphorylated by T4 Polynucleotide Kinase (NEB). The following oligos were designed and synthesized for Ago1–2 and control (CTRL) guides, respectively: Ago1–2_fw, CAC CGC GCA TCA TCT TCT ACC GCG A; Ago1–2_rev, AAA CTC GCG GTA GAA GAT GAT GCG C; CTRL_fw, CAC CGG CGA GGT ATT CGG CTC CGC G; CTRL_rev, AAA CCG CGG AGC CGA ATA CCT CGC C. For CRE-GFP vector construction, a CRE-NLS-IRES cassette from CRE-PuroR vector (Addgene, number 30205) was excised by double restriction with NotI and NdeI (NEB), filled in with T4 DNA polymerase (NEB), and inserted into a pWPXLd vector (Addgene, number 12258) which was cut with MluI and blunted as above. This resulted in a self-inactivating transfer plasmid carrying a CRE-IRES-EGFP cassette driven by the strong ubiquitous promoter EF1α.

Lentiviral vectors were produced by transient transfection of 293 T cells using 150 nM polyethylenimine (PEI) reagent (Sigma) with either PL-SIN-Nanog-EGFP, shCtrl, or shSA4 plasmids, together with the Δ8.91 packaging and a VSV-G envelope expressing plasmids [69] in a ratio of 20 μg:15 μg:5 μg, respectively, per single 100-mm dish. Transfection medium was discarded 24 h after transfection, then viral particles were collected at 48 h and 72 h, pooled and frozen at −80 °C.

For shRNA-mediated knock-down experiments, ES cells underwent two rounds of spinoculation (1 h, 1100 RCF at room temperature) [70], then transduced cells were selected in puromycin (0.75 μg/mL) for 48 h before ELA formation (Additional file 1: Figure S3a). By 0 DIV more than 90 % of cells were DsRed+, achieving a knock-down of SmarcA4 mRNA to 21.2 % of the expression level in shCtrl-infected cells (Additional file 1: Figure S3b).

For CRISPR/Cas9 experiments, cells were transduced and selected as above and maintained in ES medium (either serum and LIF or 2i plus LIF), achieving genomic editing (Additional file 1: Figure S4b) and strong down-regulation of Ago* protein levels (Fig. 6b) by 4 days after transduction. Rapamycin (100 nM; Abcam) or MG-115 (1 μM; Sigma-Aldrich) activity was tested by adding the drugs to the culture medium 48 h after transduction.

For Dicer conditional knock-out experiments, a Dicer *fl/-* ES cell line was transduced with CRE-GFP lentiviral vector, then, after 48 h, GFP-positive cells were sorted by cytofluorimetry and reseeded for culturing in ES medium. Control (not excised) cells were transduced with pWPXLd vector devoid of CRE recombinase.

### FACS analysis

Cells were detached by trypsinization, washed, and resuspended in PBS at room temperature, then analyzed with a FACSCalibur cytometer (BD Bioxciences). At least 25,000 events per sample were collected. Data were processed with Bioconductor package flowCore [71].

### HNP cell line generation

For the generation of a mouse ES cell line carrying human Nanog proximal promoter (Additional file 1: Figure S3d), E14Tg2A cells in FCS plus LIF were infected with PL-SIN-Nanog-EGFP lentiviral vector. After six passages from transduction, cells were detached by trypsinization, washed, and resuspended in PBS, 2 % FBS, and 2 mM EDTA and kept on ice. The Nanog::GFP+ population (Additional file 1: Figure S3e) was sorted on a FACS Aria II Flow Cytometer (BD Biosciences) and reseeded as a polyclonal line for amplification (HNP) to average positional effects of promoter insertion. The resulting cell line remains GFP+ after multiple passages in 2iL (data not shown) but promptly switches off fluorescence upon differentiation (Additional file 1: Figure S3f), with kinetics consistent with developmental Nanog regulation. EGFP mRNA levels correlate well with that of endogenous Nanog, as seen by real-time PCR (Additional file 1: Figure S3g).

### Nanog promoter methylation assay

For the characterization of Nanog promoter methylation status, we extracted genomic DNA with the Wizard SV Genomic DNA Purification System (Promega). DNA (500 ng) was bisulfite-treated using an EZ DNA Methylation-Lightning Kit (Zymo Research) and the region of proximal Nanog promoter (Fig. 5g) was amplified by PCR with a primer set from [72] and cloned into a pGEM-T Easy vector (Promega). For each experiment, DNA obtained from 10 to 15 different colonies transformed with plasmid was sequenced on an ABI3730 DNA analyzer (Applied Biosystems) and data were analyzed with BiQ Analyzer software [73].

### Small RNA-seq and analysis

The sequencing procedure was done using Illumina Sequencing By Synthesis technology. Total RNA (1 μg) extracted with a miRNeasy Mini kit (Qiagen) was used for library preparation (Illumina, TruSeq Small RNA Sample Prep Kit) following the manufacturer’s description. Libraries were sequenced on a miSeq (Illumina) running in 50-bp single-read mode using sequencing chemistry v3 and demultiplexed in FASTQ format using CASAVA v.1.8 (Illumina).

Library adaptors were trimmed with Trimmomatic [74] and reads were mapped to the mouse genome (NCBI37/mm9) with Bowtie [75]. miRNAs reads were annotated according to miRbase20 [76] and summarized using featureCounts [77]. Normalization was performed as reads per million (RPM) and differential expression was evaluated with the R package edgeR [78].

### In silico analysis of 3′ UTR binding

We predicted the affinity of the specific miRNA subset which is expressed in ES cells and is down-regulated during the ES–ELA transition (log_2_ mean count per million ≥5; log_2_ fold change ≤ −1; Additional file 5: Table S5) towards different mRNA families (as in Additional file 3: Table S2). For this purpose, we took advantage of the UCSF Table Browser data retrieval tool [79], which allows batch downloading of the 3′ UTR sequences of the genes of interest; when genes had several 3′ UTR variants, the longest one was taken into account. We employed the miRanda algorithm, which searches for complementarity matches between miRNAs and 3′ UTRs using dynamic programming alignment and thermodynamic calculation [80] (Additional file 5: Table S5). We set a minimum score of 140 and a maximum energy of −10 kcal/mol as thresholds. For each gene, miRNA scores were summed and the distributions of sums in different families were tested for statistically significant difference with a Wilcoxon test. 3′ UTR randomization was obtained by scrambling the 3′ UTR sequences [81]. Global miRNA–mRNA binding prediction was performed using the miRVestigator framework [82] submitting as inputs the lists of either the top Ago-released (ΔE ≤ −2) or Ago-loaded (ΔE ≥ 2) mRNAs during the ES–ELA transition. Only miRNAs with a *p* value ≤0.002 were retained in the z-score analysis.

### 3′ UTR transfection assay

The 3′ UTR of mouse Kdm2b, SmarcA4, and Dnmt3b genes were PCR-amplified from E14 ES cell genomic DNA with GoTaq (Promega) using the following primers: Kdm2b_forward, CCA GGA CAA GTA TGT AAA TAT GGA GGG; Kdm2b_reverse, GTA CAA TTG TTT ATA TAA ATC CAA CAA AGG TC; SmarcA4_forward, ACC AGA CAT TCC TGA GTC CTG; SmarcA4_reverse, CCA AGG CAA GTC CTA CTT ATT TAT TTC; Dnmt3b_forward, TTC TAC CCA GGA CTG GGG AGC; Dnmt3b_reverse, GAG AAA TAC AAC TTT AAT CAA CCA GAA AGG. The products of amplification (1035 bp for Kdm2b, 1262 bp for SmarcA4, and 1326 bp for Dnmt3b) were cloned downstream of EGFP in a pEGFPC1 expression vector (Addgene) and used in co-transfection assays together with pDsRed-Express-C1 (Addgene) and mature miRNAs (miRIDIAN microRNA Mimics, Dharmacon). Plasmid DNA and small RNA were co-transfected using Xfect polymer and Xfect RNA transfection reagents (Clontech), respectively, according to the manufacturer’s instructions. E14 cells were plated in 12 multi-well plates (4 × 10^5^/well) in ES cell medium 8 h before transfection. Cells were co-transfected in triplicate with 50 pmol of mature miRNA, 0.5 μg of 3′ UTR/pEGFPC1 vector or pEGFPC1 empty vector, and 0.5 μg of pDsRed-Express-C1, which was used as an internal reference of transfection. Both pEGFPC1 and pDsRed-Express-C1 vectors carried a CMV promoter and SV40_PA_terminator to drive constitutive expression. Four hours after transfection, ES cell medium was replaced with CDMM (ELA cells) or new ES cell medium (ES cells). EGFP and DsRed fluorescence was detected by a GloMax®-Multi + Microplate Multimode Reader (Promega) 48 h after transfection. The EGFP/DsRed fluoresence ratio was compared among different samples of transfected cells to evaluate the effect of the 3′ UTR on EGFP translation.

### Availability of data and material

Raw microarray data have been deposited in the Gene Expression Omnibus database with SuperSeries accession GSE79655 (https://www.ncbi.nlm.nih.gov/geo/query/acc.cgi?acc=GSE79655) and SubSeries accessions GSE79649 (Ago-RIP), GSE79650 (ELA-EpiSC profiling), and GSE79652 (chromatin remodeling manipulation). Raw polysome profiling and small RNA sequencing reads are available as SubSeries GSE79653 and GSE79654, respectively.

### Ethics approval

No ethics approval was required for this study.

## Additional files

Additional file 1: Figures S1–S4. Supplementary Figures 1 to 4 and corresponding legends. (PDF 3821 kb) Additional file 2: Table S1. Top RISC-released/loaded genes and top ribosome-loaded/released genes during the ES–ELA transition. (XLS 260 kb) Additional file 3: Table S2. Gene Ontology terms enriched in mRNA which are released from RISC during the ES–ELA transition; Gene Ontology terms enriched in mRNA which are loaded on ribosomes during the ES–ELA transition; Families of chromatin remodelers showing the name and mRNA NCBI accession ID of their members. (XLS 73 kb) Additional file 4: Table S3. Results of proteomic relative quantifiction of nuclear extracts from ES and ELA cells; Genes, unrelated to chromatin remodelers, with similar regulation of mRNAs in Fig. 4b–d. (XLS 24 kb) Additional file 5: Table S5. List of miRNAs downregulated during the ES–ELA transition; miRanda scores predicted for mRNAs in Fig. 4i–k. (XLS 31 kb) Additional file 6: Table S4. mRNAs that are differentially expressed during the ES–ELA transition as evaluated by microarray analysis. (XLS 148 kb)
