# Supplementary material for: RISC-mediated control of selected chromatin regulators stabilizes ground state pluripotency of mouse embryonic stem cells
Source: Genome Biol. 2016 May 6;17:94. doi: 10.1186/s13059-016-0952-x (PMC4858858; doi:10.1186/s13059-016-0952-x)
Supplement: Additional file 1: Figures S1–S4. — Supplementary Figures 1 to 4 and corresponding legends. (PDF 3821 kb) [file 13059_2016_952_MOESM1_ESM.pdf]

SUPPLEMENTAL FIGURES
Fig. S1

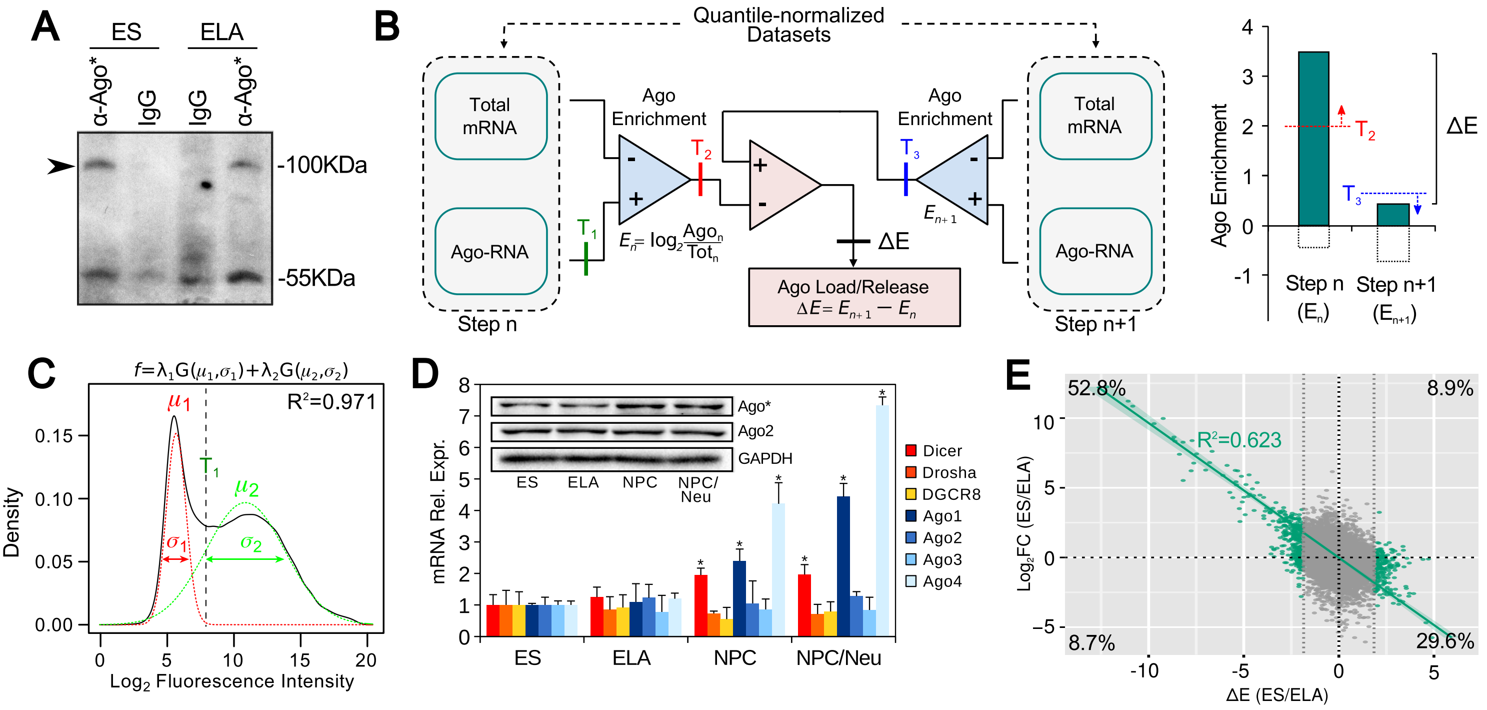

Fig. S1 - Legend

A, Western blot of protein immunoprecipitation with anti pan-Ago antibody (α-Ago*) or with total mouse IgG, in ES and ELA cells (see also for comparison Fig. S2F). Arrowead indicates Ago* proteins. IgG are large bands migrating at about 50 KDa. **B**, scheme of the experimental workflow of mRNA analysis as described in Materials and Methods. Total mRNA, or Ago-interacting RNA (Ago-RNA) isolated by Ago*-immunoprecipitation, were analyzed by gene expression microarrays. Ago enrichment (E_n) is the $\log_2$ ratio between Ago-RNA and total-RNA expression levels after quantile-normalization. T_1 is a threshold discriminating the mRNAs that are really bound to Ago from those that are not (background, see below, C). T_2 and T_3 are thresholds to evaluate mRNAs that are released (or loaded) during a step transition: released genes have $E_n \geq T_2$ and $E_{n+1} \leq T_3$, while loaded genes have $E_n \leq T_3$ and $E_{n+1} \geq T_2$. ΔE reveals the amount of mRNA loading on (or released from) Ago during a step transition. **C**, fluorescence intensity distribution (black curve) is fitted by a linear combination of two gaussian functions (dashed red and green curves, respectively) of mean μ_n, standard deviation σ_n and relative weight λ_n. T_1 is set equal to $\mu_1+3\sigma_1$, which corresponds to p=0.001 of false-positive bound mRNA. R^2 is the coefficient of determination. **D**, RT-PCR analysis of miRNA processing genes and Western blot of Ago* and Ago2 in ES cell during *in vitro* differentiation. **E**, Scatter plot shows linear regression between $\log_2$ ES/ELA mRNA expression fold-change (*y* axis) and ES/ELA ΔE (*x* axis) of green dots, reporting mRNAs with $|\Delta E| \geq 2$. Percentages indicate the fraction of mRNAs in each quadrant over total green population.

Fig. S2

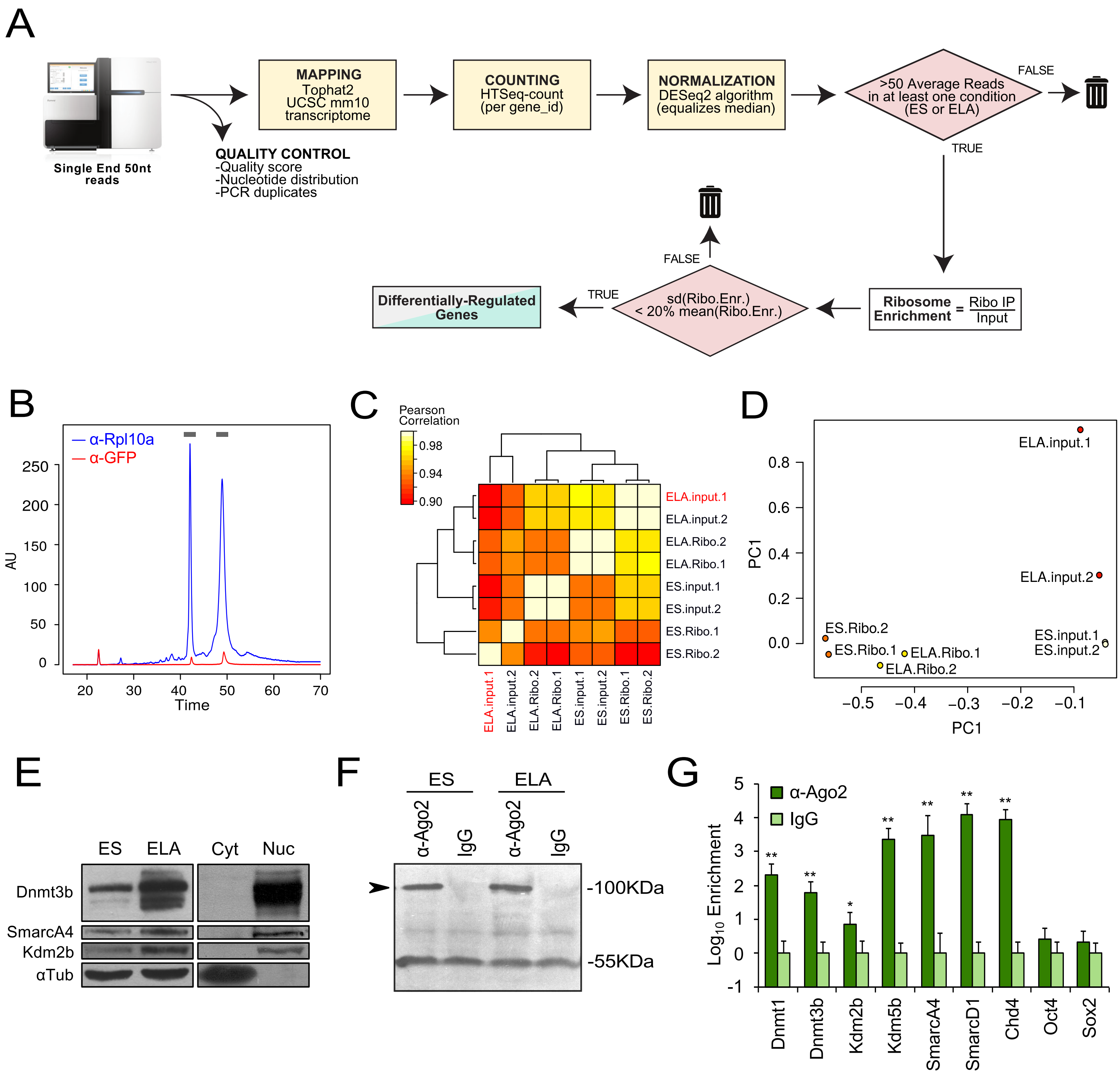
A
Single End 50nt reads
MAPPING
Tophat2
UCSC mm10 transcriptome
QUALITY CONTROL
-Quality score
-Nucleotide distribution
-PCR duplicates
COUNTING
HTSeq-count
(per gene_id)
NORMALIZATION
DESeq2 algorithm
(equalizes median)
>50 Average Reads in at least one condition (ES or ELA)
FALSE
TRUE
Ribosome Enrichment = Ribo IP / Input
sd(Ribo.Enr.) < 20% mean(Ribo.Enr.)
FALSE
TRUE
Differentially-Regulated Genes
B
α-Rpl10a
α-GFP
AU
Time
C
Pearson Correlation
0.98
0.94
0.90
ELA.input.1
ELA.input.2
ELA.Ribo.2
ELA.Ribo.1
ES.input.1
ES.input.2
ES.Ribo.1
ES.Ribo.2
D
PC1
ELA.input.1
ELA.input.2
ES.Ribo.2
ES.Ribo.1
ELA.Ribo.1
ELA.Ribo.2
ES.input.1
ES.input.2
E
ES
ELA
Cyt
Nuc
Dnmt3b
SmarcA4
Kdm2b
αTub
F
ES
ELA
α-Ago2
IgG
-100KDa
-55KDa
G
α-Ago2
IgG
Log10 Enrichment
Dnmt1
Dnmt3b
Kdm2b
Kdm5b
SmarcA4
SmarcD1
Chd4
Oct4
Sox2

Fig. S2 - Legend

A, flow-chart of Polysome profiling analysis (see Materials and Methods). **B**, Capillary electropherogram (Bioanalyzer) of RNA after pull down with antibody against Rpl10a (blue) or GFP (red). Gray bars show peaks for 18S (left) and 28S (right) ribosomal RNAs. **C,D,** hierarchical clustering of Pearson correlation (C) and PCA (D) of polysome profiling samples. **E**, Western blot of Dnmt3b, SmarcA4 and Kdm2b in total extracts of ES and ELA cells, or in cytoplasmic (Cyt) and nuclear (Nuc) extracts of ES cells. **F**, Western blot of immunoprecipitated Ago2 and IgG in ES and ELA cells. Arrohwhead indicates Ago2 protein. Mouse IgG are large bands migrating at about 50 KDa. **G**, RT-PCR analysis of mRNAs after immunoprecipitation. Values were obtained by ΔΔCT analysis using Tol2 spike-in mRNA as reference and then normalized to IgG. In **G**, *p=0.05, **p=0.01 (REST randomization test).

Fig. S3

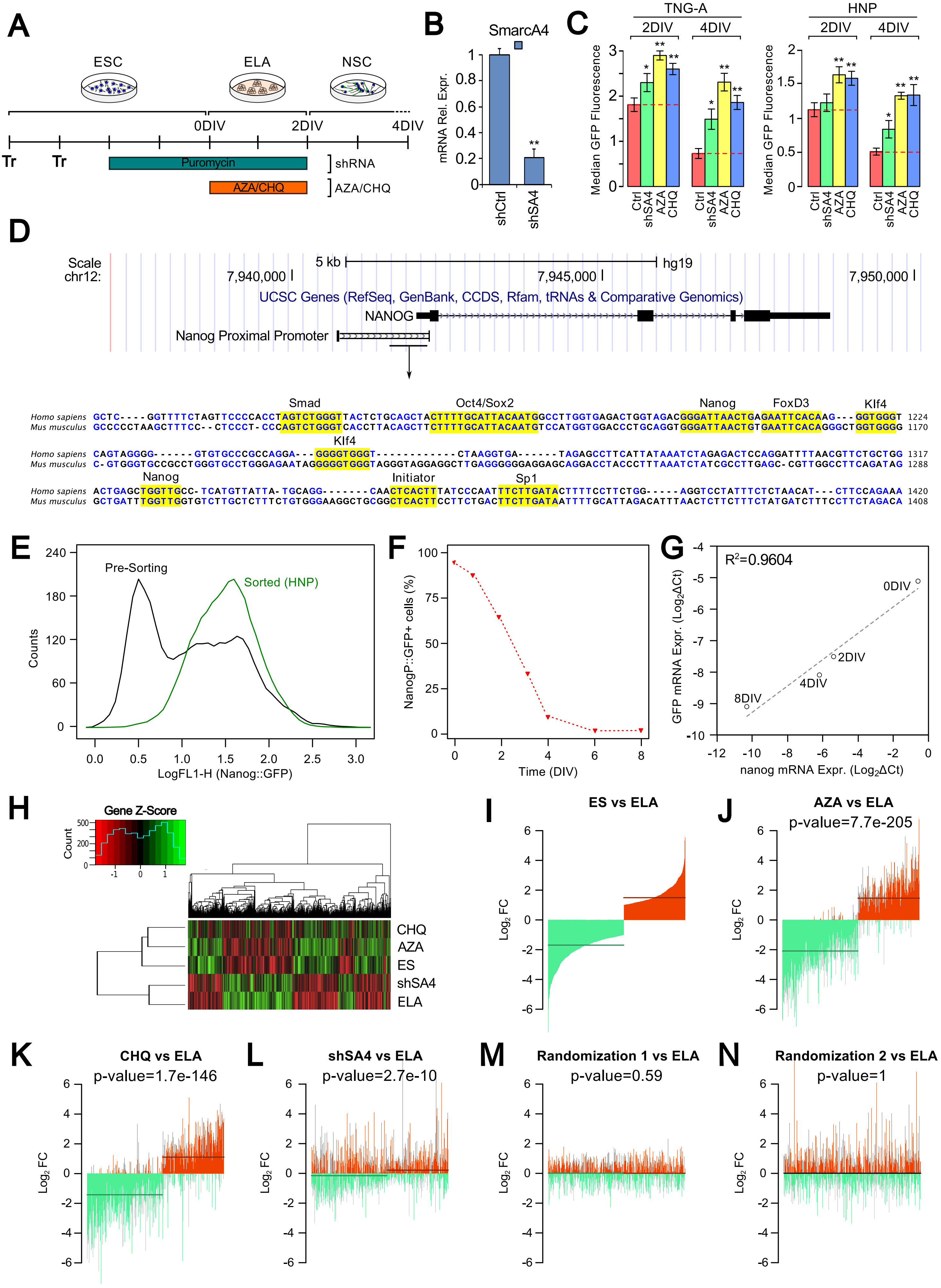
A
ESC
ELA
NSC
0DIV
2DIV
4DIV
Tr
Tr
Puromycin
AZA/CHQ
shRNA
AZA/CHQ
B
SmarcA4
mRNA Rel. Expr.
shCtrl
shSA4
C
TNG-A
HNP
2DIV
4DIV
Median GFP Fluorescence
Ctrl
shSA4
AZA
CHQ
D
Scale
chr12:
5 kb
hg19
7,940,000
7,945,000
7,950,000
UCSC Genes (RefSeq, GenBank, CCDS, Rfam, tRNAs & Comparative Genomics)
NANOG
Nanog Proximal Promoter
Smad
Oct4/Sox2
Nanog
FoxD3
Klf4
Homo sapiens
Mus musculus
Initiator
Sp1
E
Pre-Sorting
Sorted (HNP)
Counts
LogFL1-H (Nanog::GFP)
F
NanogP::GFP+ cells (%)
Time (DIV)
G
R²=0.9604
0DIV
2DIV
4DIV
8DIV
GFP mRNA Expr. (Log₂ΔCt)
nanog mRNA Expr. (Log₂ΔCt)
H
Gene Z-Score
Count
CHQ
AZA
ES
shSA4
ELA
I
ES vs ELA
Log₂ FC
J
AZA vs ELA
p-value=7.7e-205
K
CHQ vs ELA
p-value=1.7e-146
L
shSA4 vs ELA
p-value=2.7e-10
M
Randomization 1 vs ELA
p-value=0.59
N
Randomization 2 vs ELA
p-value=1

Fig. S3 - Legend

A, scheme of the protocol for functional interference with chromatin remodelers: for lentiviral-mediated shRNA delivery, ES cells (ES) underwent two subsequent rounds of transduction (Tr) followed by puromycin selection, mantained also during embryoid body (ELA) formation. Chemical inhibitors 5-Azacytidine (AZA) and 5-Carboxy-8-hydroxyquinoline (CHQ) were added directly in ELA medium; DIV: days of *in vitro* differentiation. **B**, RT-PCR analysis showing mRNA expression of SmarcA4 in shCtrl- and shSA4-transduced cells. Values are relative to β-actin mRNA expression, with shCtrl level normalized to 1. **C**, flow cytofluorimetric analysis of the TNG-A and HNP cell lines, 2 and 4 days after 2iL withdrawal (2DIV and 4DIV, respectively); shSA4, cells transduced with shSA4 lentiviral vector and cultured in control minimal medium; AZA and CHQ, control cells cultured in 5-azacytidine (50 nM) and 5-Carboxy-8-hydroxyquinoline (100 μM), respectively. **D**, Nanog proximal promoter region and sequence conservation. The scheme represents the region of human nanog promoter driving the reporter vector PL-SIN-Nanog-EGFP, as obtained by the UCSC genome browser (http://genome.ucsc.edu/). Kb, kilobases; chr, chromosome. The lower part shows the alignment of a portion of human and mouse sequences: although the overall degree of homology is not high, the core regulatory modules (yellow boxes) are conserved. **E**, green fluorescence distribution (FL1-H) of cells transduced with lentiviral vectors carrying PL-SIN-Nanog-EGFP, before (black curve) and after (green curve) cell-sorting of the EGFP-positive population. **F**, time-course of EGFP-positive cell percentage during differentiation (DIV: days of *in vitro* differentiation). **G**, Diagram showing the correlated expression of EGFP (y axis) and nanog (x axis) mRNA at different DIV. Values are expressed as $\log_2\Delta Ct$ of RT-PCR assay; R^2 is the coefficient of determination. **H**, Hierarchical clustering analysis of mRNA expression of ES cells (ES) and of ELA cells in control medium (ELA) or treated as indicated. Inset shows the distribution of genes (count) with respect to their z-score. **I-N**, bar plots indicating the distribution of $\log_2$ fold changes ($\log_2$ FC in *y* axis) of mRNA expression of genes whose expression is mostly changed between ES and ELA cells (see Materials and Methods). Lines show the median fold change of up- (red) or down- (green) regulated genes. p-value refers to sign test. Randomization 1-2, datasets of values randomized as explained in Materials and Methods. AZA- and CHQ-treated cells show a gene expression fold change profile almost identical to that of ES cells. shSA4 cells, although showing a more different profile, have a significant tendency toward ES cell identity. In B,C, *p=0.05, **p=0.01 (B, REST randomization test; C, Student's t-test).

Fig. S4

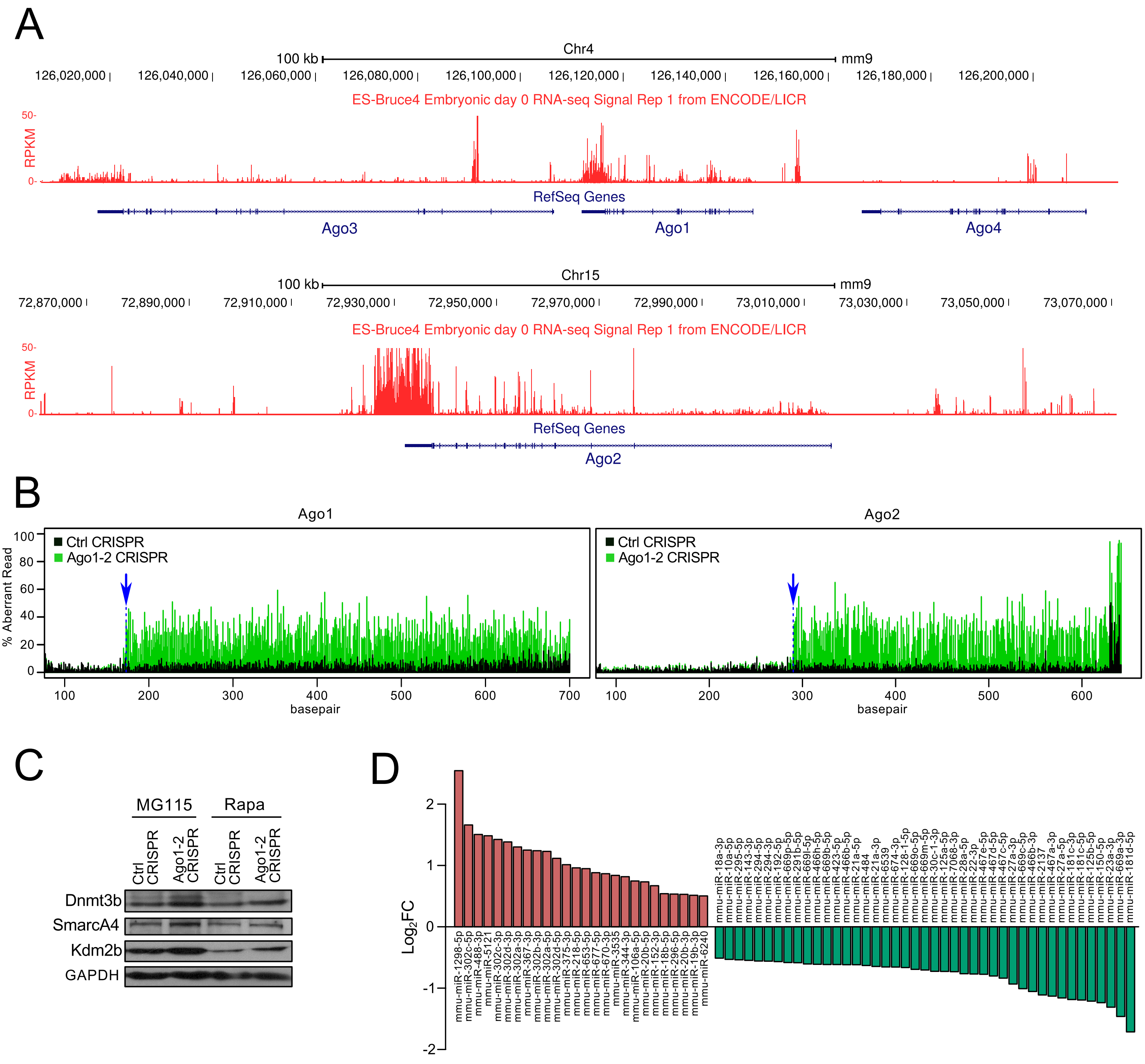
A
Chr4
100 kb
mm9
126,020,000
126,040,000
126,060,000
126,080,000
126,100,000
126,120,000
126,140,000
126,160,000
126,180,000
126,200,000
ES-Bruce4 Embryonic day 0 RNA-seq Signal Rep 1 from ENCODE/LICR
RPKM
50-
0-
RefSeq Genes
Ago3
Ago1
Ago4
Chr15
100 kb
mm9
72,870,000
72,890,000
72,910,000
72,930,000
72,950,000
72,970,000
72,990,000
73,010,000
73,030,000
73,050,000
73,070,000
ES-Bruce4 Embryonic day 0 RNA-seq Signal Rep 1 from ENCODE/LICR
RPKM
50-
0-
RefSeq Genes
Ago2
B
Ago1
Ago2
% Aberrant Read
Ctrl CRISPR
Ago1-2 CRISPR
100
80
60
40
20
0
100
200
300
400
500
600
700
basepair
C
MG115
Rapa
Ctrl CRISPR
Ago1-2 CRISPR
Ctrl CRISPR
Ago1-2 CRISPR
Dnmt3b
SmarcA4
Kdm2b
GAPDH
D
Log2FC
2
1
0
-1
-2
mmu-miR-1298-5p
mmu-miR-302c-5p
mmu-miR-488-3p
mmu-miR-5121
mmu-miR-302c-3p
mmu-miR-302d-3p
mmu-miR-302a-3p
mmu-miR-367-3p
mmu-miR-302b-3p
mmu-miR-302a-5p
mmu-miR-302d-5p
mmu-miR-375-3p
mmu-miR-218-5p
mmu-miR-653-5p
mmu-miR-677-5p
mmu-miR-670-3p
mmu-miR-3535
mmu-miR-344-3p
mmu-miR-106a-5p
mmu-miR-20b-5p
mmu-miR-152-3p
mmu-miR-18b-5p
mmu-miR-296-5p
mmu-miR-20b-3p
mmu-miR-19b-3p
mmu-miR-6240
mmu-miR-18a-3p
mmu-miR-10a-5p
mmu-miR-295-5p
mmu-miR-143-3p
mmu-miR-294-5p
mmu-miR-294-3p
mmu-miR-192-5p
mmu-miR-669p-5p
mmu-miR-291b-5p
mmu-miR-669l-5p
mmu-miR-466h-5p
mmu-miR-669b-5p
mmu-miR-423-5p
mmu-miR-466b-5p
mmu-miR-21a-5p
mmu-miR-484
mmu-miR-21a-3p
mmu-miR-6539
mmu-miR-674-3p
mmu-miR-128-1-5p
mmu-miR-669o-5p
mmu-miR-669m-5p
mmu-miR-30c-1-3p
mmu-miR-125a-5p
mmu-miR-7068-3p
mmu-miR-28a-5p
mmu-miR-22-3p
mmu-miR-467e-5p
mmu-miR-467d-5p
mmu-miR-467c-5p
mmu-miR-27a-3p
mmu-miR-669c-5p
mmu-miR-466b-3p
mmu-miR-2137
mmu-miR-467a-3p
mmu-miR-27a-5p
mmu-miR-181c-3p
mmu-miR-181c-5p
mmu-miR-125b-5p
mmu-miR-150-5p
mmu-miR-23a-3p
mmu-miR-669a-3p
mmu-miR-181d-5p

Fig. S4 - Legend

A, Gene plots show RNA-seq profiles of Argonaute genes in mouse ES cells as reported in dataset ES-Bruce4 (UCSC Genome Browser; [1]). The distribution of reads per kilobase per million on *y* axis (RPKM) indicates that only Ago1 and 2 are significantly expressed. We obtained similar evidence by the analysis of our gene expression datasets with both ES and ELA cells (data not shown). **B**, Aberrant reads (*y* axis) along the genomic sequence of Ago1 (left panel) or Ago2 (right panel) in cells transduced with control CRISPR lentiviral vector (dark green) or Ago1-2 CRISPR lentiviral vector (light green). This analysis was carried by Sanger sequence trace decomposition [2]**.** **C**, Western blot of Dnmt3b, SmarcA4 and Kdm2b in control (Ctrl) or Ago1-2 CRISPR ES cells cultured in the presence of inhibitors of proteasome (MG115; 1uM) or mTOR pathway (rapamycin, Rapa, 100nM). **D**, Selection of top-regulated miRNAs ($|\log_2$ FoldChange$|\geq 1$) at ES/ELA cell transition. FC, fold change.

SUPPLEMENTAL REFERENCES

1. Mortazavi A, Williams B a, McCue K, Schaeffer L, Wold B: **Mapping and quantifying mammalian transcriptomes by RNA-Seq.** *Nat Methods* 2008, **5**:621–628.

2. Brinkman EK, Chen T, Amendola M, van Steensel B: **Easy quantitative assessment of genome editing by sequence trace decomposition**. *Nucleic Acids Res* 2014, **42**:e168–e168.
